# Polyphenols: Secondary Metabolites with a Biological Impression

**DOI:** 10.3390/nu16152550

**Published:** 2024-08-03

**Authors:** Ecem Bolat, Sümeyye Sarıtaş, Hatice Duman, Furkan Eker, Emir Akdaşçi, Sercan Karav, Anna Maria Witkowska

**Affiliations:** 1 Department of Molecular Biology and Genetics, Çanakkale Onsekiz Mart University, Canakkale 17000, Türkiye; ecemmbolatt@gmail.com (E.B.); sumeyyesaritas@stu.comu.edu.tr (S.S.); hatice.duman@comu.edu.tr (H.D.); furkan.eker@stu.comu.edu.tr (F.E.); emirakdasci@gmail.com (E.A.); 2 Department of Food Biotechnology, Bialystok Medical University, 15-089 Bialystok, Poland

**Keywords:** secondary metabolite, health, biological activity, nutrition

## Abstract

Polyphenols are natural compounds which are plant-based bioactive molecules, and have been the subject of growing interest in recent years. Characterized by multiple varieties, polyphenols are mostly found in fruits and vegetables. Currently, many diseases are waiting for a cure or a solution to reduce their symptoms. However, drug or other chemical strategies have limitations for using a treatment agent or still detection tool of many diseases, and thus researchers still need to investigate preventive or improving treatment. Therefore, it is of interest to elucidate polyphenols, their bioactivity effects, supplementation, and consumption. The disadvantage of polyphenols is that they have a limited bioavailability, although they have multiple beneficial outcomes with their bioactive roles. In this context, several different strategies have been developed to improve bioavailability, particularly liposomal and nanoparticles. As nutrition is one of the most important factors in improving health, the inclusion of plant-based molecules in the daily diet is significant and continues to be enthusiastically researched. Nutrition, which is important for individuals of all ages, is the key to the bioactivity of polyphenols.

## 1. Introduction

Today, as developing countries increase, life expectancy in these countries is also increasing. However, this evolving and changing lifestyle is also leading to a proliferation of age-related diseases, including cancer, diabetes, metabolic disorders, heart diseases, hepatitis, and neurological disorders. For some diseases, there are still a lack of early detection tools or a lack of suitable treatment, influencing researchers to focus on preventive treatments. In this context, researchers are looking for treatments by focusing on diet and nutritional models, such as the Mediterranean or Asian diets. Such dietary patterns may reduce the risk of aging-related diseases brought about by changing lifestyles [1]. Mediterranean and Asian diets, which are generally plant-based diets, are based on plant sources including fruits, vegetables, seeds, herbs, and legumes [2]. These diets emphasize the potential role of polyphenols, the natural constituents of plants that indicate their nutritional value, in the prevention of aging and disease, and emphasize healthy lifestyles [3]. Therefore, more research is needed on the importance of plant polyphenols for human health and their use in early the treatment or prevention of diseases.

Polyphenols are water-soluble, also known as plant-derived natural products, and have a molecular weight between 500 and 4000 Da. They are abundant from originally plant foods including vegetables, grains, beverages, and fruits [4]. Plant foods include these plant-derived products as secondary metabolites [5]. Therefore, plant species synthesize secondary metabolites to protect themselves against biotic-like bacteria, fungi, or insects and abiotic stress like environmental stress, free radicals, or metabolic disorders [6,7]. Polyphenols, natural plant phenolic compounds, are a complex category with more than 8000 different types. Based on the diverse criteria, they are classified according to comprise one or more phenolic ring structures with single or multiple substituent groups, which are hydroxyl (OH) bounding [1]. These structural arrangements result in the diversity of polyphenols. Depending on the number of phenolic rings and the structures that bring these rings together, polyphenols can be roughly divided into five categories: phenolic acids, flavonoids, stilbenes, lignans, and tannis [8]. Due to these structural features and various biological interactions, polyphenols are known for their versatile biological properties, including antioxidant, anti-inflammatory, anticancer, antibacterial, and anti-aging properties [9,10]. Therefore, polyphenols have been shown to have great potential in the treatment of various diseases such as metabolic, cardiovascular, neurodegenerative, and cancer diseases [9,11]. This review article investigates the main categories of polyphenols evaluated as secondary metabolites, their extraction processes, and their characterization. The bioavailability of these components and various effects on health were presented based on research. In addition to these effects, the benefits of polyphenol consumption in infant, mother, child, adult, elderly, and athlete nutrition are discussed.

## 2. Polyphenols’ Categories

**Figure 1:**
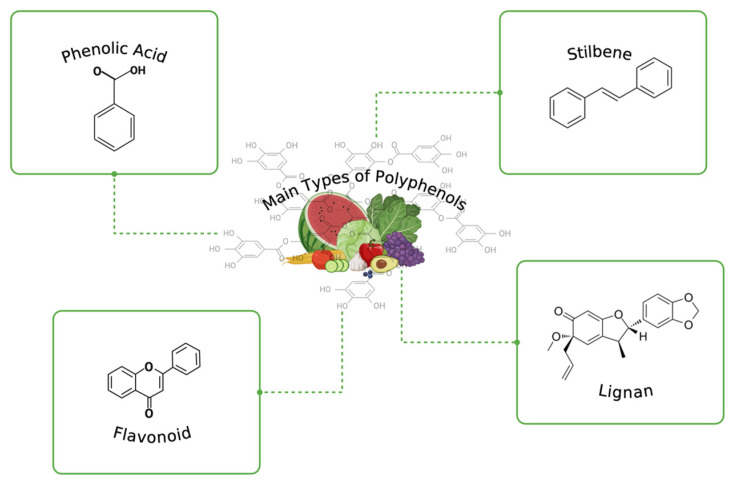
Main categories of polyphenols.

Polyphenols demonstrate a basic phenolic structure, and these natural compounds are distinguished or categorized depending on their chemical structures, in particular the amount of the aromatic ring, bounded molecule on the ring, and ring-binding element structures determine the classification (Figure 1).

### 2.1. Phenolic Acids

The structural characteristic of phenolic acid possesses one phenolic ring unlike the other. This class resembles phenolic compounds, which are structured with one carboxylic acid group with one or more hydroxyl derivatives. Thus, it is mainly associated with amides, esters, and glycosides [12]. There are two subgroups of phenolic acids, hydroxybenzoic and hydroxycinnamic acid. Benzoic acid-derived hydroxybenzoic acid has a C6-C1 carbon core, while cinnamic acid-derived hydroxycinnamic acid is present in plants as a simple ester with glucose or quinic acid [13]. Phenolic acid is found in a variety of foods, especially herbs, vegetables, cereals, fruits, legumes, and beverages [14]. These compounds demonstrate antioxidant activity by the donation of hydrogen atoms, so phenolic acids exhibit antioxidant activity [14]. In addition, phenolic acids are important for their ability as medicinal compounds in diverse disease treatments, in particular diabetes, cardiovascular disorders, cancer, and neurodegenerative problems [15,16]. Their key structural form, including their aromatic ring, number and position of hydroxy moieties (-OH), and unsaturated chain, enables them to show many biological activities, especially anticancer activity [17].

### 2.2. Flavonoids 

Flavonoids are the most researched and known class of polyphenols. Flavonoid’s fundamental structure is composed of two aromatic rings with a three-carbon chain with one oxygen, which is a generated oxygenated heterocycle. This class of polyphenols can be classified into a number of subgroups depending on the oxidation state of the central carbon. For this purpose, flavonoids are divided into six different classes which are named as flavanols, flavonols, isoflavones, flavanones, flavones, and anthocyanidins [14,18]. Flavonoids, known as a secondary metabolite in some plant structures (fruits and seeds), are important for their ability to provide color, taste, and aroma. This diverse group of polyphenols includes a wide range of different varieties, and is among the most well-researched class in plant science [14]. Flavonoids, which play a complementary role in plant metabolism, facilitate growth, and also function in protective mechanisms [19,20]. Moreover, this class has an important function in reducing radical oxygen species (ROS) formation in plants under stress, as they can regulate the activity of ROS. Thus, it contributes to a variety of potential bioactive activities that improve health in humans, including antioxidant, anti-inflammatory, and protective effects against cardiovascular diseases, and neuroprotective, anticancer, and anti-aging roles [21,22,23,24,25].

### 2.3. Stilbenes

This class of polyphenols is determined by two aromatic rings connected by a methylene bridge and a significant class of nonflavonoid phytochemicals [14]. Stilbenes are also a kind of phenylpropanoid, and it is characterized by 1,2-diphenylethylene core [26]. The most popular representative compound is resveratrol, which is rich in red wine and naturally present in grapes and peanuts [18,27]. As the resveratrol compound is rich in red wine, there is a hypothesis about how to prevent chronic diseases [28]. Moreover, anti-inflammatory and antioxidant abilities are possible for resveratrol compounds [18,29]. Additionally, it was reported that resveratrol compounds are possible to act as a protector of the preservation of wine [30]. This other study researched this situation, and the composition and quantity of stilbene in wines has been defined as a quality indicator [31]. 

### 2.4. Lignans

Lignans, the other class of polyphenols, resemble phenolic acids structurally [18]. However, in the diphenolic group structure of lignans, the carbon–carbon bond is there. Polymerization of two units of phenylpropane units produces this natural compound, and is extensively distributed in seeds, roots, stems, and leaves of plant [32]. Additionally, eight subgroups of lignans are there according to their carbon structure, cyclization pattern, and way of oxygen incorporation into the molecule structure. Furans, furofurans, arylnaphthalenes, aryltetralins, dibenzylbutanes, dibenzylbutyrolactones, dibenzocyclooctadienes, and dibenzybutyrolactols are the subgroups of lignans. Furthermore, the position of the oxygen atom affects the categorization of lignans [14]. In addition to lignans properties, they are mostly found in the vegetables (fiber-rich plants), legumes, and cereals. Foods rich in lignan are associated with various pharmacological effects on human health, and therefore the consumption of a lignans-rich diet may be beneficial to human health [33]. An especially favorable effect of lignans is anticancer activity [34]. Lignans may control or prevent cancer by many different mechanisms, and so up to now, there are some in vivo studies about the anticarcinogenic effect of pure lignans [35,36,37,38,39,40,41]. Additionally and apart from that, lignans possess anti-inflammatory, antioxidant, anti-menopausal (protective role form cardiovascular, bone, and other psychological disorders), and antimicrobial properties [42,43,44].

## 3. Methods for Extraction and Determination of Polyphenols

Polyphenols have different structures of phenolics, and thus they possess different chemical and physical characteristics. These characteristics feature many significant extraction methods for their availability [45]. Moreover, the understanding and decision of the proper extraction are important because the chemical structure of the substance, the particle size of the sample, and also the presence of the other substances to be used interact with each other. Apart from this, extraction efficiency is also a point to be considered in order to obtain phenolic compounds. Several parameters such as ambient temperature, pH value, correct solvent selection, solvent and sample ratio, number of repeated extractions, and extraction time affect extraction efficiency [46]. Furthermore, although it is still difficult to analyze polyphenols, it is very valuable for the literature to search for sensitive and accurate methods not only for extraction, but also for estimating polyphenols. New data that can be brought to the literature on the identity and dosage of polyphenols are the first step in evaluating the health benefits of polyphenols [47].

### 3.1. Types of Most Common Extraction Methods

- Ultrasound-Assisted Extraction (UAE)

The ultrasound-assisted extraction, which is a very effective method for the extraction of phenolic compounds, gives an accurate result with a high yield in a short time duration [48]. Ultrasonic radiation possesses higher than 20 kHz frequencies, thus making the extraction of inorganic and organic compounds easy to perform with liquid solvents [49]. This widely used method is known to be environmentally friendly, not only reducing the extraction time, but also the volume of solvent and energy input [50]. Ultrasonic waves damage the cell wall of plants, increasing its permeability by stimulating the phenomenon called cavitation, which leads to many expansion and contraction movements on the surface of a solid sample [51]. This allows the liquid solvent to reach the cell matrix and facilitate the free release of water-soluble compounds from the plant [50]. Additionally, in recent years, this method has been used in studies on the polyphenols of pecan nutshell, randia monantha, olive pomace, mango seeds karnels, and pine needles, with emphasis on the optimization of this method and the polyphenol extraction after its use and the antioxidant-antifungal effects of these polyphenols [48,50,51,52].

- Microwave-Assisted Extraction (MAE)

Microwave-assisted extraction is a green extraction method that is suitable for obtaining and isolating polyphenols from samples including herbs, plants, and plant-based materials. Water is generally used as the solvent in this method, and is considered to be a faster, cheaper, and more reliable compared to other methods. Since the heat released during the extraction process affects the yield of the targeted polyphenols, optimization of the conditions that are affecting the extraction is significant for this method [53]. Solvent type and ratio, microwave power, and extraction time are the most important conditions affecting extraction efficiency [54]. These conditions must be to optimize and maintain for the sample to obtain maximum yield. The solvent type can be water or ethanol, and the ratio can be adjusted for the sample. In the research studies conducted, the biological properties of the polyphenols obtained as a result of extraction were investigated, and their antimicrobial, antioxidant, anticancer, and other activities were determined [55,56]. With the microwave-assisted extraction method, polyphenols were commonly extracted from waste and by-products [55,57]. In this way, in line with the zero waste policy, polyphenols obtained from waste and by-products, including peels, pomace, and leaves, can be used for nutraceutical and pharmaceutical purposes [54,58,59].

- Ultrasound–Microwave-Assisted Extraction

The ultrasound–microwave-assisted extraction technique, which is a novel approach combining ultrasonics and microwaves, minimizes the extraction time, uses lower volumes of solvents, and results in higher yield of bioactivated compounds compared to the other two MAE and UAE techniques [60]. It is a powerful method in which plant samples are heated with microwaves and bioactive compounds are removed from the cell using dielectric mechanisms and the simple penetration of the cell wall is facilitated by ultrasound [61]. In a recent study, MAE and UAE were compared, and it was shown that MAE exhibited higher polyphenol content and antioxidant activity [62]. However, the extraction process was evaluated to be a shorter time. The use of UAE demonstrated that less energy was consumed and that the process was more environmentally friendly. When the results were evaluated, it was thought that the extraction process and the yield of extracted polyphenols would be improved when ultrasound- and microwave-assisted extraction were combined. In another study conducted by Ma et al., polyphenols extracted from mangosteen peels using two different methods, which are ultrasound–microwave-assisted extraction and enzyme–ultrasound-assisted extraction [61]. Both methods demonstrated similar total phenolic content; however, enzyme–ultrasound-assisted extraction exhibited higher polyphenol yield than ultrasound–microwave-assisted extraction. Additionally, extracted polyphenols from both methods demonstrated potential application in the pharmaceutical and functional food additives industries.

- Supercritical Fluid Extraction (SFE)

Another extraction method is supercritical fluid extraction (SFE), which is performed with two steps: first, soluble bioactive compounds are extracted from the plant cell matrix with the action of supercritical fluid (SCF); then, with gas depressurization, the extracted bioactive compounds are dissociated from SCF, where fluids are converted into the gaseous phase [63,64]. The SFE method is based on the use of SCF, which can be formed when the temperature (40–80 °C) and pressure (10–35 MPa) enhance higher than its critical value [63]. With this technique, the use of toxic solvents (hexane, methanol, methyl tert-butyl ether, dichloromethane) can be reduced, and therefore the safety of using the technique can be improved [65]. Therefore, SFE is one of the so-known green methods, and it widely uses CO_2_, CH_3_, C_2_H_6_, C_2_H_6_O, C_3_H_8_, C_6_H_6_, and NH_3_ for the gas depressurization step [66]. Additionally, by not exposing bioactive compounds to air and light during the process, degradation of these compounds can be prevented, and the possibility of contamination of the sample with an impure solvent can be much reduced compared to other methods [67]. Additionally, in recent years, this method has been used in studies of the polyphenol of *Ailanthus excelsa*, *Dunaliella salina*, and chestnut shells [64,65,68]. As distinct from these studies, a research study demonstrated that the detection of bioactive compounds of moringa oleifera, which posses anti-yeast, anti-diabetic, wound-healing activities [69].

- Other extraction methods

In addition to UAE, MAE, and SFE technologies, there are a few other techniques for the extraction of phenolic compounds. One of these techniques is subcritical water extraction (SCWE), also called superheated water or hot liquid water extraction [70]. Based on this method, when the temperature reaches between 100 and 347 °C and special pressures (range of up to 220 bar), the water is in subcritical form and retains its liquid forms [71]. Under these subcritical conditions, the intermolecular hydrogen bonds of water will be broken and the dielectric constant will be decreased, and thus so does the adjustment of the temperature and the pressure affect of the water dielectric constant and the whole extraction process. Additionally, compared to the SFE method, it can be more cost effective because it uses water instead of organic solvent [72]. Like the other methods, SCWE also gives high quality results, low process duration, and is eco-friendly [73]. In recent years, there are some studies that show this method has been used with phenolic and natural compounds of Cocoa pod husks, and Saffron tepals were observed with this technology [71,73,74,75]. Another technique for the extraction of phenolic content is pulsed electric field (PEF), a non-thermal electric field processing [76], which uses directly high electric current between the two electrodes, as if they were sandwiched, and with two different sub-methods depending on the number of pulses: batch (100–300 V/cm) and continuous (20–80 kV/cm) [77]. Electric charges accumulate on the membrane of cells that are sandwiched between two electrodes. Due to charge accumulation, a high amount of transmembrane potential develops, thus increasing membrane permeability and facilitating the release of phenolic compounds out of the cell [77]. The medium, degree of electroporation, and physical-chemical characteristic of the plant cell and tissues can impact the effectiveness of PEF [78,79]. In recent years, polyphenols of liposomes loaded with Green tea, Laurel leaves, Cannibis, and *Phyllanthuse emblica* were extracted with PEF and their antioxidant and anti-inflammatory activities and optimization of the PEF has been demonstrated [80,81,82,83]. Lastly, accelerated solvent extraction (ASE), also called pressurized liquid extraction (PLE) [84], uses extensively organic solvents in the presence of nitrogen, and extract analytes need to be solid or semi-solid. Also, under the influence of high temperature and pressure, it quickly penetrates into the plant cell membrane and accelerates the extracellular release of phenolic compounds without disrupting their structure [66]. This new green extraction method serves lower solvent consumption and energy, and higher efficient extract yields. Furthermore, another feature that distinguishes it from other methods is that it can be automated. With a small intervention, this is more reproducible and preserves the efficiency of the extraction process [85]. In recent years, phenolic extraction of strawberry and onion peel ASE was performed to evaluate the antimicrobial-antibiofilm properties and also optimize the ASE [85,86].

### 3.2. Types of Most Common Quantification Methods for Polyphenols

- Spectrophotometric Assays

One of the simple technologies for the determination of plant phenolic compounds is spectrophotometry assay [87]. In this technology, the Folin–Denis and Folin–Ciocalteu methods were commonly to estimate whole phenolic plant materials. In recent years, the total phenolic content of broken bone twigs and phenolic composition and antioxidant activity of tanacetum parthenium was detected using these two methods [88,89]. These both good screening methods involve some reagents such as tungsten and molybdenum for chemical reduction, which is the basis of the procedures [90]. Furthermore, total flavonoids, condensed tannin, and phenolic quantification can be performed using colorimetric methods by mixing them with AlCl3 provided, estimated at the 410–423 nm range of wavelengths [91]. An additional important thing is that anthocyanins constitute detection, and it can be measured with spectrophotometry and performed in weak acidic media between the wavelength range of 490 to 550 nm [92]. Colorimetric techniques for detecting this type of phenolic plant material are straightforward and economical. However, they only give an estimate of phenolic compounds at a specific minimum concentration and cannot measure individual compounds [66]. Nevertheless, these techniques can be a good choice for determining a large number of plant bioactive compounds in a faster and economically cheaper way. In recent years, anthocyanins detection has been performed using red poppy as a colorimetric sensor [93]. Also, other determinations of anthocyanin research has been conducted, and plant samples were both wild elderberry and grape (grape juice) [94,95].

- Gas Chromatography (GC)

Gas chromatography (GC) is also used for the quantification and determination of some kinds of polyphenols, namely flavonoids, phenolic acids, and tannins [96]. Based on this method, the gas phase is translocated within the column by a mobile phase (gas carrier) like He, N2, and H2. Additionally, this method can be performed using gas–solid absorption or gas–liquid partitioning, where the stationary phase is a non-volatile liquid [97], and also an ionization flame detector is used for this method [98]. Silica capillaries (columns) with a length of 30 m, an inner diameter of 25–32 μm, and a stationary phase particle size of 0.25 μm are commonly used in this technique [66]. Recently, the co-linking of GC with a mass spectrometer has also been increasingly used, as it provides greater selectivity and sensitivity [98]. It is highly sensitive to observe the breakdown pattern of plant bioactive compounds and then determine their composition by comparing these observations with mass spectrometry data of the compounds [97]. In recent years, phytochemical screening of Kleenex wild has been performed using GC-mass analysis [99]; also, other studies in which the polyphenol contents of sonneratia caseolaris fruits has been estimated using GC and their antimicrobial properties is demonstrated [100], and fast-growing leaves have been analyzed and optimized to determine their bioactive compound content with GC-mass [101].

- High-Performance Liquid Chromatography (HPLC)

High-performance liquid chromatography (HPLC) is still the most preferred analytic tool for the determination and quantification of phenolic compounds. Usually, after the purification of phenolic compounds, purified compounds are put on the C18 column (stationary phase) [102], which is an instrument of HPLC and is used for photodiode array detectors and acidified polar organic solvents. In the wake of advancing technology, chromatographic fingerprint analysis was discovered and recognized as an innovative, faster method for the identification and characterization of herbal medicines. Chromatographic fingerprint profiles can be described as an authentic material of the plant, identifying the species of a specific plant and distinguishing it from other species [66]. Moreover, the sensitivity or efficiency of HPLC can be changed based on the pre-concentration of phenolics of plant extracts, purification of phenolics, and also mobile phase and column selection. During the determination of phenolics, especially arranging the pH of the mobile phase is so important, because the irregular pH value causes the ionization of the phenolic compounds that are in the process of being determined. The choice of the appropriate column can usually be determined with a column for different phenolic classes and particle sizes, depending on the polarity. However, more advanced HPLC techniques can use innovative types of columns for different particle sizes [47]. The duration of HPLC takes between 10 and 150 min, and if the analysis of HPLC takes more time to determine phenolic compound, a constant temperature is needed [103]. In recent years, phenolic compounds, metabolomic profiling, the antioxidant-antimicrobial properties of apple pomace, lysimachia nummularia, grape juice, and acacia were determined with HPLC [104,105,106,107].

- Other quantification methods

In addition to spectrophotometric assays, GC, and HPLC methods, there are a few other methods for the detection of plant bioactive materials, and these are paper chromatography (PC), capillary electrophoresis (CE), and supercritical fluid chromatography (SFC). First of all, PC is a thin-layer and simple method for detection of especially tea leaf bioactive compounds [87]. A research study that demonstrated using high-performance thin-layer chromatography can be suitable for the detection of caffeine from green tea leaves [108]. In addition to this study, the biological properties of medicinal plant extracts like antioxidant, anti-inflammatory, and antimicrobial assessment are performed via PC. A study concluded that the flavonoids or presence of fatty acids can show these biological properties [109]. However, PCs are less utilized when compared to HPLC and GC methods. In continuation, another method is capillary electrophoresis (CE), which is a high-quality yield method by using narrow capillary columns containing ion solutions. This technique is good for the detection of low–medium molecular weight and charged plants’ bioactive material, and also it is rapid, effective, and a lower volume of plant samples and process reagents are needed [110]. There are different types of CE, such as capillary electrochromatography (CEC), micellar electrokinetic (MEKC), capillary zone electrophoresis with UV (CZE), and with mass spectrometry (MS), commonly applied among diverse types of CE [66]. In recent years, the application of CE to tobacco analysis and free sulfur dioxide determination in wine and cider were performed [111,112]. Also, the analysis of cassine and spectaline in the senna spectabilis was obtained using CZE with indirect UV detection [113]. Lastly, supercritical fluid chromatography (SFC) is a novel technology and is generally used to analyze versatile plant material [114]. When compared to other HPLC and GC methods, this technique possesses high efficiency, high quality results, short process duration, and is eco-friendly [66]. The column structure based on fully porous particles below −2 μm or superficially porous particles below −3 μm is one of the highlights of this technique [115]. These new analytical methods are used in recent research studies, and both demonstrate the detection of softwood lignans and isomeric forms of urolithin glucuronides [116,117].

## 4. Bioavailability of Polyphenols

The bioactivity of polyphenols depends on their ability to reach an action position. The bioavailability of polyphenols refers to the amount of nutrients derived from digested, absorbed, and metabolized polyphenols [118,119]. The bioavailability of polyphenols is influenced not only by their transmembrane capacities, but also by their structural compositions, size, and previous diet, sex, intestinal microflora, and nutrient matrix [120,121]. In addition, polyphenols interact with gut microbiota stains, which affect the status of molecules and lead to modification. Also, there are more than one denaturing conditions for polyphenols [117], including pH, enzymes, heat, light, and oxygen, which further lead to low bioavailability and limit the utilization of polyphenols for bioactive agents [122]. Polyphenols exhibit variations in bioavailability due to their different forms, including esters, glycosides, or polymers [118]. In the study [95] in which the chemical and functional characterization of polyphenols in the seeds of *Cannabis sativa* L. was carried out, it was shown that phenolic compounds had a bioavailability of 142.39% after the digestion process, while flavonoid class molecules were shown to have less bioavailability with a rate of 29.47% [123]. Moreover, the study investigating the in vitro gastrointestinal digestive effect on the stability and bioavailability of polyphenols, derived from wild and commercial Mexican blackberries (*Rubus* spp.), showed that bioaccessible phenolic molecules of wild blackberries may have higher bioavailability and bioactivity in the human body compared to commercial ones [119]. Researchers have demonstrated that the stability of polyphenols is better in organic solvents or water compared to cell culture medium, where it is significantly worse. This indicates that the presence of polyphenols in the human biological environment is easily degradable, potentially resulting in low bioavailability and the inability to exhibit biological activity [124]. 

Typically, most dietary polyphenols are hydrolyzed in the small intestine or colonic microflora and then methylated and replaced by glucuronide and sulfation metabolites by liver and other organs [120]. The backstairs indications of absorption of polyphenols from polyphenol-rich foods through the intestinal barrier is demonstrated by the increasing antioxidant ability in the plasma medium [125]. Especially, consumption of red wine, tea, blackcurrant, and apple juice can affect the antioxidant ability in the plasma medium [125]. For more direct indications of bioavailability of polyphenols, it has been shown by measuring concentrations in plasma and urine after consumption of pure compounds or foodstuffs known to contain the compound of interest [126]. Taking polyphenols provides a positive health effect; however, the low ability to reach the desired action position of polyphenols in the body makes it difficult for positive health effects to occur, because of their low reabsorption (only 5 to 10 percent by the small intestine) and rapid transformation along with excretion [121]. 

A study aimed to show the antioxidant and anti-tumor activity of crude (BBCP) and purified (BBPP) blueberries extracted in vivo and in vitro [127]. In the result of the study, the in vitro experiments of BBPP possessed stronger antioxidant activity; however, BBCP had higher antioxidant activity in vivo experiments. It was hypothesized that results may also be linked with bioavailability, which occurs in in vivo experiment systems. A study was carried out aiming to maintain the bioavailability of polyphenols while designing sport nutrition products combining milk protein and plant polyphenols in sport nutrition [128]. The study showed that milk–blackberry blends, especially those made with whole full cream milk, preserved anthocyanins during in vitro digestion and increased their bioavailability. In a different study, the bioavailability of the total polyphenol compounds of coffee silver skin extracts analyzed was evaluated using a simulated gastrointestinal (GI) digestion and colonic fermentation [129]. It was suggested that antioxidant and bioavailability increased during GI, and that during colonic fermentation, these antioxidant components may reach the biological action site and exert potential health effects. Additionally, another study about the effect of heat treatment on phenolic compounds of sweet potato (*Ipomoea batatas* L.) showed that antioxidant activity and phenolic content increased after heat treatment [130]. The presented results provide a better understanding of the effect of heat treatment on bioactive compounds in sweet potatoes, and may thus contribute to the improvement of product processing technology by both maintaining and enhancing bioavailability. At the same time, encapsulation of polyphenols with liposomes and nanoparticles can support bioavailability. In this way, encapsulated polyphenols may show more effective bioactivity [122].

### Bioavailability of Encapsulated Polyphenols or Polyphenols Covered with Liposomes or Nanoparticles and Their Effect of Functionalities

Encapsulation is essentially a drug- or food-ingredient-loaded delivery system. In the food industry, this delivery system traps active ingredients, protects them from degradation during the storage–processing phases, and enhances their bioactivity by facilitating their delivery to the site of action (tissue or organ) [122]. Polyphenols have not been fully utilized in functional foods and food supplements due to their physicochemical properties such as poor oral bioaccessibility matrix, poor solubility in GI fluids, molecular transformations in the GI tract, and low permeability through epithelial cells [131,132,133,134]. Therefore, there are different technologies that enhance the bioavailability of polyphenols, and two of them are more prominent, nanoencapsulation and liposomal encapsulation. To deliver any bioactive compound to diverse sites of action requires a small particle size. Since the diameters’ range of nanoencapsulation are between 10 and 1000 nm, it possesses to enhance bioavailability, protect from denaturing conditions, and easily deliver the precision targeting of the bioactive compound [135]. Another one is liposomal encapsulation, which is a superior technology for sensitive bioactive compounds of hydrophobic and hydrophilic molecules and ideal for nutrient bioavailability to reach full efficacy. The lipid- and water-oriented system provide higher surface permeability and solubility; thus, this technology permits bioactive compounds to find a target accurately. Additionally, the lipophilic complex provides easy absorption in the intestine and protection from unfavorable intestinal interaction or degradation during absorption and digestion statement [136]. Because of these advantages of two different technologies, studies have been carried out, showing the potential to increase the bioavailability of polyphenols and thus improving the bioactivity. 

A liposome-encapsulated grape seed extract was used to demonstrate the anti-aging, skin-brightening, and hydrating effect on human skin cells [137]. The result of the study promoted youthful appearance, resulting in more soluble, lighter-colored formulations, and suitable for a broad range of skincare products [138]. Another study was conducted to improve bone wound healing, with liposome form of gallic acid, in rats [139]. In this study, rats were divided into four different groups: a negative control, a positive control, a gallic acid powder group, and a gallic acid liposome group. The most improvement was observed in the gallic acid liposome group, while the least improvement was observed in the negative control. Also, the occurrence of infection was the highest in the negative control group and the lowest in the gallic acid liposome group. In this context, it was interpreted that the bioavailability of gallic acid polyphenol encapsulated with liposomes increased, and thus more effective bioactivity could be formed. Other studies, which are about nanoliposome-encapsulated polyphenols from different plant sources, show and increase the antimicrobial bioactivity by enhancing their bioavailability [140,141,142]. One of these studies, extract of *Rheum ribes* plants was encapsulated with nanoliposome for performing as a novel phytogenic antibiotic against *Escherichia coli* (*E. coli*) in mice [141]. The study concluded that 10 mg TPC/kg of encapsulated polyphenols improves the health parameters in mice higher than the nonencapsulated one. Another similar study used a phenolic-rich fraction from different plant *Alcea rosea* to dietary phytobiotic role on mice [142]. The aim of this research is to evaluate the antibacterial and potential health-promoting activity of nanoliposomes’ phenolic-rich fraction against *E. coli.* All results demonstrated that the 10 mg TPC/kg encapsulated polyphenols were more effective in improving the health parameters of mice than the unencapsulated ones. Another research includes nanoliposome-encapsualted phenolics from *Achillea millefolium* plant and its antimicrobial function against *Campylobacter jejuni* (*C. jejuni*) infection [140]. The same quantity of nanoencapsulated polyphenols improved food intake, liver function, and antioxidant status of mice. Also, more decreased the population of *C. jejuni* in infected mice with nanoencapsulated treatment than the non-encapsulated ones. Therefore, the nanoliposomes polyphenols could be considered phytobiotic against this type of infection. Additionally, a different study aimed to treat the same bacterial infection in mice, but aimed to do this effect with a particular plant, *Artemisia aucheri* [143]. The researchers found similar results with this study.

An in vivo rat model study demonstrated that liposomes-encapsulated ferulic acid protected from induced oxidative liver damage [144]. As a result of the study, encapsulated ferulic acid has antioxidant effects by reducing cytotoxicity induced by CCl4 in vitro in rats. Furthermore, administration intravenously effectively decreased CCl4-induced hepatotoxicity, ROS generation, and tissue damage of the rat livers. Another animal model study demonstrated liposomes-encapsulated *p*-coumaric acid (CA) could inhibit osteoclast formation and bone resorption in rheumatoid arthritis challenged rats [145]. As a result of the study, CA potentially inhibits bone erosion and prevents the loss of calcium. An animal study includes broiler breeder roosters aimed at improving post-thawed sperm quality by ellagic acid-loaded liposomes [146]. In the general result of the study, 1 mM ellagic acid-loaded liposomes demonstrated a positive effect on sperm quality. Antioxidant status of the thawed sperm was improved.

In a study about the photodynamic therapy of cancer with polyphenols of green tea, according to the study, nanoparticles of green tea polyphenols possessed a more inhibitory role on cancer cell proliferation and enhanced the apoptosis rate of cancer cells than normal non-nanoparticles [147]. This means that the study demonstrated that the bioavailability and anticancer bioactivity of green tea polyphenols is increased by the nanomedicine. Moreover, it demonstrated the anti-tumor activity of rosmarinic acid-loaded silk fibroin nanoparticles on HeLa and MCF-7 cells. Actually, rosmarinic acid (RA), known as polyphenols, possess antioxidants, antimicrobials, etc., and bioactive roles. In this study, nanoparticles are considered advantageous for maintaining the bioavailability of polyphenols. Hence, the antitumor activity of polyphenol RA is increased by enhancing the solubility of RA using nanoparticles [148]. Ren et al. conducted a different study for enhancing anti-tumor activity of curcumin nanoparticles [149]. Curcumin is a powerful phenolic compound because of their biological effects on the body, including anti-tumor, antioxidant, anti-inflammatory effects. However, it has limitations in utilizing curcumin, such as its instability and limited oral bioavailability. Therefore, in Ren’s study, encapsulated bioactive substances using nanoparticle strategy were used, and then curcumin was encapsulated into pea protein isolate using a pH-driven method. As a result of the study, pea protein isolate–curcumin nanoparticles with very high loading rates and improved water solubility were obtained. A different study was performed to prepare strong antioxidative and anticancer therapeutic nanoparticles from Tea polyphenols using an amino acid-induced ultrafast procedure [150]. To prepare a therapeutic nano agent with epigallocatechin gallate (EGCG), an antioxidant from especially green tea, a simple and fast method using five kinds of amino acids, arginine, lysine, glycine, leucine, and glutamic acid was used. As a result of the study, arginine and lysine are ended within 50 s with a very short induction reaction. The prepared nanoparticles exhibited potent antioxidant capacity ten times higher than the commonly applied nanoparticles, and had therapeutic effects on tumors, especially as confirmed by in vitro and in vivo evaluation. Another study used *Punica granatum* (*P. granatum*) (Pomegranate) for green synthesizing of silver nanoparticles and demonstrated its antimicrobial role [151]. According to the study, green synthesis of silver nanoparticles that are a polyphenols-rich fraction was performed, and the fraction demonstrated antimicrobial activity against *Bacillus subtilis* (*B. subtilis*), *Staphylococcus aureus* (*S. aureus*), and *Sarcina lutea.*

In general, the bioavailability of encapsulated polyphenols could increase; therefore, their bioactivity, solubility, or permeability could enhance. However, not enough studies are available, and further research is needed, which demonstrates that other different biological effects of polyphenols can be enhanced by encapsulation.

## 5. Health Benefits of Polyphenols

**Figure 2:**
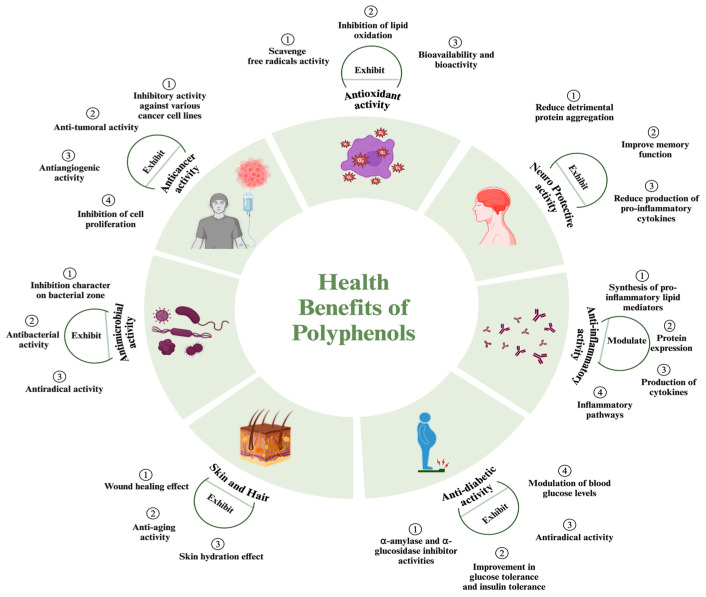
Representation of health benefits of polyphenols.

Since various foods and beverages including fruits, vegetables, tea, and wine have rich polyphenol content, including these foods in the diet is an effective way to benefit from the health-promoting properties of polyphenols (Table 1) (Figure 2) [152,153,154,155,156]. Polyphenols exhibit antioxidant, anti-inflammatory, antimicrobial, antidiabetic, anti-aging, anti-tumor/anticancer, and cytotoxic activities [157,158,159,160]. Thanks to these roles, they can reduce the risk of chronic diseases and alter the treatment of diseases [161,162]. They have also been shown to have positive effects on cardiovascular health and cognitive functions, which can prevent neurodegenerative conditions [163]. 

### 5.1. Antioxidant Activity

The antioxidant activity of polyphenols is one of the most extensively researched properties [164,165,166,167]. A key property of polyphenols is actually their reduction or prevention from reactive oxygen species (ROS), which is known as their antioxidant role [168,169,170]. Polyphenols have capability to scavenge ROS, which have deleterious effects on human health [171,172]. Therefore, polyphenols exhibited a prevention role on stress or age-related diseases as well as skin deterioration in humans [4]. Because of their structural conformation, polyphenols react with these active molecules mainly in three different mechanisms, which are Hydrogen Atom Transfer (HAT), Single Electron Transfer (SET), and Transition Metal Chelation (TMC) [173,174]. For the HAT mechanism, the structure of polyphenols, including the functional phenolic group, poses hydrogen-donating capacity by breaking the O-H bond in hydroxyl molecules to a free radical [173]. This mechanism is characterized by bond dissociation enthalpy (BDE). With the occurrence of a lower BDE value of the O-H bond, there is expected to be higher activity, for instance, when a free radical R removes hydrogen atoms from an antioxidant ArOH (R +ArOH = ArO + RH) [175,176]. The SET mechanism is characterized by ionization potential (IP), and molecules with low IP value are indicated as high activity by their electron-donating capability, such as, a single electron–proton abstraction from ArOH (R +ArOH = R-AROH+ = RH + ArO) [175]. Polyphenols anions poses the specific ability for the chelation of heavy metals; therefore, in the TMC mechanism, because of the deprotonated hydroxyl formation, heavy metals can be chelated and then produce a proton (ArOH = ArO + H+) [177]. These three mechanisms demonstrate the capacity of polyphenols’ antioxidant role to protect human health from reactive species [174].

In addition, various polyphenols exert distinct effects on antioxidant activity [178,179]. Certain polyphenols, such as quercetin, have been shown to possess particularly potent antioxidant properties [180]. Polyphenols, known for their antioxidant properties, have been extensively investigated in both in vitro and in vivo studies [181,182]. These compounds, known for their health-promoting effects, also demonstrate a protective role against various diseases [183,184]. In this study, conducted by Kukhtenko et al., the pharmacological potential and medical applications of *Rhododendron tomentosum* were explored, with a focus on its polyphenol content [185]. High-performance thin-layer chromatography analysis detected the presence of RA, caffeic acid, chlorogenic acid, rutin, and quercetin in the plant extracts. Subsequently, following the 2,2-diphenyl-1-picrylhydrazyl (DPPH) radical scavenging activity test, it was revealed that these polyphenols exhibited significant antioxidant activity.

Menhem et al.’s study was performed on a DPPH assay to demonstrate the Zhourat plant’s antioxidant activity [186]. Their different solvent extraction was performed after determining the total phenolic acid. Each plant solvent extraction was tested, and free radical scavenging activity of them was calculated. Except for two different extracts, generally, water/ethanolic extracts demonstrate higher antioxidant activity than the pure water or ethanolic extracts [186]. In another study conducted by Bashmil et al., the antioxidant capacity of Australian-grown bananas was tested [187]. According to the results of the study, it has been proven that the super unripe form bananas have the ability to scavenge more free radicals compared to ripe form bananas, and grown bananas tested with more than one different free radicals have been shown to have antioxidant capacity. It was also confirmed that the antioxidant properties of bananas vary depending on which type of polphenols they contain and the position and number of phenolic rings these polyphenols have [187]. Moreover, medical plant *Chamerion angustifolium* L. (*C. angustifolium* L.) (Rosebay Willowherb Holub) was analyzed for its antioxidant role [167]. In the solid-state fermentation antioxidant analysis of leaves of *C. angustifolium* L., the antioxidant potential of the leaves of different fermentation types and times, as well as non-fermented leaves, varied. It was showed that antioxidant activity in *C. angustifolium* L. leaves decreased significantly after 24 h in aerobic and anaerobic fermentation. However, the antioxidant activity of aerobic and anaerobic fermented leaves increased after 48 h compared to unfermented leaves. 

An original article conducted by Janarny et al. is about antioxidant ability of *Fabacea* family edible flowers [188]. In this study, the determination of total phenolic content, total flavonoid, and total anthocyanin contents was performed. Then, the antioxidant scavenging activity of *Fabacea* family flowers was completed against nitric oxide (NO) and hydrogen peroxide. All of the results of the study exhibited the potential antioxidant role of these family’s flowers. Moreover, another research study completed by Bobkova et al. is about the antioxidant role of coffee, and according to the study, the total antioxidant capacity was performed as determined using free radical scavenging [189]. The results of the study demonstrated that coffee samples have an antioxidant role and also that a growing region of the coffee samples affects the polyphenol content of the coffee. Alsaud et al. reported on the free radical scavenging activity (DPPH assay) and ferric reducing antioxidant activity (FRAP assay) of *Leptospermum scoparium* (Manuka leaves) [190]. According to the results of the study, the antioxidant activity of ethanolic extract is quite higher than the other deep eutectic solvents (DES) extracts, and for the FRAP method, the usage of other DES4 extracts of manuka leaves showed a superior antioxidant capacity.

In conclusion, polyphenols exhibit antioxidant effects through various mechanisms, such as scavenging free radicals and enhancing antioxidant enzyme activity [191,192,193].

### 5.2. Anti-Inflammatory Activity

Polyphenols, characterized by their unique aromatic ring structures and hydroxyl groups, exhibit strong regulatory effects on various inflammatory pathways [194,195]. In vitro and in vivo studies demonstrate the ability of polyphenols to inhibit the expression and activity of major pro-inflammatory mediators, including nuclear factor-κB (NF-κB), a transcription factor that plays a central role in regulating the inflammatory response [196,197]. Polyphenols appear to inhibit the activation of NF-κB, a transcription factor that has an important role in regulating the expression of pro-inflammatory genes [198,199]. By inhibiting NF-κB activation, polyphenols can effectively reduce the production of inflammatory cytokines and enzymes [198]. They can suppress the production of pro-inflammatory lipid mediators, particularly by modulating the activity of enzymes involved in the inflammatory process, thus exhibiting anti-inflammatory effects [197,200]. Additionally, polyphenols have the ability to modulate immune cell function [201]. They affect the activity of macrophages, lymphocytes, and dendritic cells in order to modulate immune cell function [202,203]. Furthermore, it appears that polyphenols may play a critical role in the recruitment and migration of immune cells by modulating the expression of adhesion molecules and chemokines [198,204]. Additionally, synergistic interactions between different polyphenols may also support anti-inflammatory activity [205]. In a study aimed at determining the anti-inflammatory properties of polyphenols extracted from parsley (*Petroselinum crispum*), coriander (*Coriandrum sativum*), and celery (*Apium graveolens*), parsley was found to have the highest total polyphenol content, followed by celery and coriander [206]. When evaluating the activity of scavenging NO, a free radical produced in high amounts during various inflammatory conditions, parsley polyphenols exhibited the highest activity. To assess the capacity of plant extracts to prevent the structural degradation of proteins, their ability to prevent protein denaturation was evaluated, with parsley showing the highest anti-denaturation potential. Lastly, the membrane stabilization effect, which evaluates the potential of plant extracts to stabilize erythrocyte (red blood cell) membranes and investigate their anti-inflammatory properties, revealed that parsley extracts had the highest potential.

Berries contain rich polyphenol materials that enable the understanding and characterization of the anti-inflammatory properties of polyphenols [207,208]. A study conducted by Kim et al. aimed to investigate the anti-inflammatory and antimicrobial activities of polyphenols obtained from the roots and unripe fruits of the black raspberry plant [209]. A series of studies aimed to determine whether black raspberry polyphenols affect cytokine production, nitrite formation, prostaglandin E2 (PGE2) production, and mRNA levels of inflammatory enzymes. The results demonstrated that black raspberry root polyphenols significantly suppressed NO and PGE2 production in lipopolysaccharide (LPS)-stimulated RAW264.7 cells in a dose-dependent manner. Additionally, it was observed that the production of pro-inflammatory cytokines decreased significantly in a dose-dependent manner with black raspberry root polyphenols compared to unripe fruit polyphenols in LPS-stimulated RAW264.7 cells. Similarly, root polyphenols significantly reduced nitric oxide synthase (iNOS) and cyclooxygenase-2 (COX-2) mRNA levels in LPS-stimulated RAW264.7 cells. Furthermore, the antimicrobial activity of polyphenols against various pathogenic bacteria was evaluated. Black raspberry root polyphenols exhibited potent antimicrobial effects against pathogenic bacteria such as carbapenem-resistant *Acinetobacter baumannii*, methicillin-resistant *S. aureus* (MRSA), and *Bacillus anthracis*.

In a study conducted by Peng et al., the aim was to obtain polyphenol extract (HPE) from huangjiu, a traditional Chinese rice wine, and to evaluate the anti-inflammatory effects of this extract [210]. The anti-inflammatory effect of polyphenol extracts isolated and purified from huangjiu were examined in RAW264.7 macrophage cells stimulated with LPS. The extract effectively reduced NO production and downregulated the expression of inducible iNOS. It also reduced the production of pro-inflammatory cytokines (TNF-α, IL-6, and IL-1β). The downregulation of iNOS expression was associated with the suppression of NF-κB translocation to the nucleus and the inhibition of the phosphorylation of Inhibitor of κB and mitogen-activated protein kinase (MAPK) family proteins (p-38, Erk 1/2, and JNK). Similarly, a study aimed at examining the antioxidant and anti-inflammatory properties of polyphenols derived from the *Ilex latifolia* (*I. latifolia*) plant evaluated the effects of these polyphenols in an LPS-induced inflammation model [211]. The study demonstrated that polyphenols modulate NO production in LPS-stimulated RAW 264.7 macrophage cells. Moreover, they were found to inhibit the production of pro-inflammatory cytokines (TNF-α, IL-1β, and IL-6) in a dose-dependent manner. Furthermore, it was shown that polyphenols dose-dependently reduce iNOS and COX-2 mRNA expression in LPS-stimulated macrophages. Additionally, polyphenols were observed to modulate the phosphorylation of MAPK family proteins (ERK and JNK) in LPS-stimulated macrophages and inhibit NF-κB activation.

A study aimed to determine the phytochemical compositions of polyphenols extracted from the bark of *Libidibia ferrea* and *Parapiptadenia rigida* plants, as well as the leaves of *Psidium guajava* (*P. guajava*) plant, and to examine their microbial activity and anti-inflammatory properties [212]. The in vitro experiments included the Agar-Well Diffusion Test and Determination of Minimum Inhibitory Concentration. *S. aureus*, *Staphylococcus epidermidis*, *Enterococcus faecalis* (*E. faecalis*), *E. coli*, *Salmonella enteritidis*, and a methicillin-resistant *S. aureus* strains were used for this purpose. The results indicated that polyphenol extracts obtained from the three plants exhibited activity against Gram-positive bacteria, although to a lesser extent against Gram-negative bacteria, demonstrating their effectiveness. Conversely, polyphenol extracts from *P. guajava* exhibited the largest zones of inhibition in agar diffusion tests compared to extracts from other plants. The anti-inflammatory effect was evaluated using the Carrageenan Peritonitis Test. Following the administration of various doses of the aforementioned plant extracts to mice, carrageenan was injected into their abdominal cavities to induce an inflammatory response. The results revealed a significant reduction in inflammation upon treatment with plant extracts. However, in the assessment of pain response through the analgesic activity test and Acetic acid coil reflex test, the plant extracts did not exhibit any significant effect on pain reduction in mice. Consequently, the study suggests that the mentioned plant extracts hold potential for the treatment of inflammatory diseases.

Fermentation has a significant impact on bioactive compounds in plants and foods [212,213]. A recent study conducted by Sim et al. aimed to examine the changes in phenolic compounds and their anti-inflammatory effects resulting from the complex fermentation of *Maclura tricuspidata* and *Pyrus montana* Nakai plants with *Aspergillus shirousamii* (koji), yeast, and lactic acid bacteria [214]. When the results of the study were evaluated, it was found that the content of phenolic compounds increased with the duration of complex fermentation, as indicated by the analysis of phenolic compounds and flavonoids in the products. Moreover, the DPPH and ABTS radical inhibition abilities of the fermented products also increased. Subsequently, the ability of the products obtained after complex fermentation to inhibit NO production was tested on LPS-stimulated RAW 264.7 cells. The results revealed that complex fermentation products significantly suppressed NO production starting from the 6th day. Furthermore, the anti-inflammatory effect of complex fermentation on cellular mechanisms was examined through Western Blot Analysis. The analysis of iNOS, COX-2, and TNF-α protein expression indicated suppression of stimulated cells. In conclusion, the study demonstrates that the anti-inflammatory effect is enhanced after complex fermentation.

Overall, studies demonstrate that polyphenols extracted from various plants, algae, and similar products play a role in alleviating inflammatory conditions and exhibit significant potential for anti-inflammatory properties [215,216,217]. Additionally, it has been revealed that it has the potential for protection against metabolic diseases [218]. 

### 5.3. Antimicrobial Activity

A molecule or a substance’s ability to break down and decelerate the proliferation or growth of microorganisms, in particular bacteria, viruses, parasites, and fungi, is known as antimicrobial activity [219,220]. To make provision for microbial diseases, antimicrobial compounds are extensively used in different areas like the medicine, agriculture, or food industries [221,222]. Similar to their other biological properties, phenolic compounds in plant extracts form the basis of antimicrobial activity [223,224,225,226]. Many polyphenols demonstrate antimicrobial activity by disrupting the cell structure and cell membrane and interfering with enzymatic cellular processes [18,227]. The presence of carboxyl groups in these phenolic compounds and the configuration of sub molecules on the benzene ring are important factors determining the antimicrobial activity of polyphenols [228]. In addition, for antimicrobial activity, the amount of carboxyl, hydroxyl, and ester groups in phenolic compounds are significant [229]. These groups promote the inhibition of microbial growth by ensuring the interaction between polyphenols and microbial cells [230]. On the other hand, polyphenols can contribute to antimicrobial activities by interfering with enzymatic and intracellular mechanisms [231,232]. They achieve this by disrupting the functioning of enzymes necessary for the survival of microorganisms, thereby inhibiting their growth [233].

A research study conducted by Angelis et al. demonstrated the protective antiviral role of polyphenols mixtures and micronutrients (A5+) on influenza A and SARS-CoV-2 infection [234]. In this study, it was demonstrated that polyphenols are resveratrol, which means having an antiviral role against respiratory virus infection. Therefore, polydatin was used in this study as a precursor for resveratrol, and also A5+ was used to inhibit infection forming. For SARS-CoV-2 infection, viruses’ strains were treated with A5+ and resveratrol, and then it was shown that the replication of SARS-CoV-2 infection reduced. For influenza A virus, again the viruses’ strains were treated with both, and it was shown that the necessary replicative viral proteins and IL-6 production of influenza A virus were inhibited.

Singh et al. performed another study that was about polyphenols as natural antiviral drugs against the SARS-CoV-2 virus [235]. This in silico analysis study aimed at the inhibition of RNA-dependent RNA polymerase (RdRp) of SARS-CoV-2 and prevention of RNA replication. According to the result of the study, molecular binding dynamics of eight different polyphenols were demonstrated to potentially inhibit the RdRp of SARS-CoV-2. Therefore, these polyphenols were exhibited as potential antiviral molecules. Pizzo et al. (2019) performed an antiviral and an antimicrobial assay of *Prunus dulcis* L. (Almond) against two different microorganisms, *S. aureus* and herpes simplex virus type 1 (HSV-1) [236]. As the result of this antimicrobial assay of almonds, complete inhibition by polyphenols was achieved with an amount of 0.62 mg/mL. Moreover, the conclusion of an antiviral assay is that 0.4 mg/mL amount of polyphenols of almonds reduced the expression of viral proteins and viral DNA accumulations. A research study conducted by Park et al. showed that the ethanolic extract of *Aronia melanocarpa* (*A. melanocarpa*) has an antiviral activity with the help of its polyphenol and flavonoids contents [237]. Even 0.0625 mg samples isolated from *A. melanocarpa* demonstrated a high seasonal anti-influenza role, and inhibited virus surface proteins with 70 percent of influenza viruses such as H1 and H3. Pagliarulo et al. performed an antibacterial analysis of *Punica granatum* L. (Pomegranate) against the bacterial growth of *S. aureus* and *E. coli* [238]. In this research, pomegranate juice was obtained from their fruits, and then ethanolic polyphenol extraction was obtained by adding 50% ethanol/water (*v*/*v*). Particularly, pomegranate juice was characterized by phenolic-containing anthocyanins. Different concentrations of pomegranate extracts (1, 2, 4, 8, 10, and 20 mg/disk) were evaluated on cultured bacteria stains. As a result of research, pomegranate juice extracts antagonize the growth and survival of cultured stains. Antimicrobial activity of these extracts was proved. Menhem et al. performed a study about antimicrobial properties of Zhourat plants using a disk diffusion assay [186]. For this assay, food-borne pathogens were tested, including two Gram + bacteria, *S. aureus* and *Bacillus cereus*, and two Gram-bacteria, *E. coli* and *Pseudomonas aeruginosa* (*P. aeruginosa*). The determined total phenolic compounds (polyphenols) of Zhourat demonstrated antibacterial activity on these bacteria. The inhibition zone diameter was diverse between different polyphenol extracts and bacterial species. Some extracts showed no effects against any of these bacteria, whereas others showed no effects against one of the bacteria.

In a study published in 2017, the total phenolic and flavonoid contents, as well as the antioxidant and antimicrobial properties of extracts from four types of Chinese tea—namely, Black Tea Flowery Broken Orange Pekoe, Broken Orange Pekoe, Red Dust, and Green Tea—were examined [239]. The study revealed that green tea is richer in total phenolic and flavonoid compounds compared to the other teas. The tea samples were evaluated for their antioxidant properties using the phosphomolybdenum and FRAP methods. As a consequence of study, green tea demonstrated higher antioxidant activity compared with the other tea samples. The antimicrobial activity of the teas was tested against *Shigella dysenteriae*, *Shigella boydii*, *Vibrio cholerae*, *Salmonella typhi*, *Salmonella paratyphi*, *Klebsiella pneumoniae*, and *E. coli* using agar well diffusion and minimum inhibitory concentration methods. The results indicated that green tea exhibited higher antimicrobial activity than the other varieties of tea.

Studies have shown that the microbial properties can be affected by the use of different extraction techniques and solvents in the extraction of polyphenols from materials [225,227]. In a study conducted by Chaudhry et al., the effects of the extraction method and different solvents on extraction were investigated [240]. For this purpose, maceration and ultrasound-assisted extraction methods, traditional extraction methods, were used. Simultaneously, three different solvents—ethanol, methanol, and acetone—were tested at concentrations of 25%, 50%, 75%, and 100%. Regarding the amount of polyphenols obtained from banana peels, it was found that a higher amount of polyphenols is obtained using the ultrasound-assisted extraction technique compared to the maceration method. It has also been shown that ethanol provides a higher extraction efficiency compared to other solvents used. However, it was observed that the concentration of the solvent used had a significant effect on the amounts of polyphenols obtained. It was revealed that ethanol extracts showed higher antioxidant activity based on the DPPH radical scavenging test. On the other hand, banana peel extracts at different concentrations were applied to *S. aureus, P. aeruginosa, E. coli*, and *Saccharomyces cerevisiae* microorganisms using the antimicrobial activity agar disk diffusion method. When evaluating the region formed around the proliferation areas of microorganisms, called the inhibition zone, it was revealed that ethanol-containing extracts have an antimicrobial effect compared to others.

Polyphenols bound to indigestible fibers in the small intestine can exert beneficial health effects by releasing phenolic compounds after microbial fermentation in the large intestine. Therefore, fermentable fiber foods in the daily diet may provide various health benefits through the release of phenolic compounds that support the growth of beneficial gut microbiota and may act as prebiotics [241].

Although the antimicrobial effects of phenolic compounds have been demonstrated in various studies, it is interesting how these effects change after the gastrointestinal digestion process [242]. In a study conducted in 2022, the aim was to determine the antioxidant activity of grape pomace extracts, the effects of the gastrointestinal digestion process on phenolic components, and the impact of these extracts on the growth of microorganisms [243]. The study revealed that the digestion process can modulate the free radical scavenging capacity of phenolic compounds [243]. Antimicrobial activity was evaluated by assessing the effects on the growth of probiotic and pathogenic microorganisms, including *Lactiplantibacillus plantarum*, *E. coli*, *Bacillus megaterium*, and *Listeria monocytogenes* (*L. monocytogenes*). The antimicrobial activity of polyphenols was determined by assessing their effects on the growth of these microorganisms. The results showed that polyphenols obtained from grape pomace increased the growth of probiotic bacteria and reduced the growth of pathogenic bacteria. On the other hand, another study aimed at evaluating the digestive behavior and antimicrobial effects of polyphenols derived from the *Hibiscus sabdariffa* (*H. sabdariffa*) plant in the human digestive system, it was demonstrated that polyphenol compounds obtained from the plant were rapidly released and secreted in the digestive system [244]. This suggests that polyphenols may be readily accessible for absorption in the upper intestines. When assessing their antioxidant properties, it was revealed that the polyphenols exhibited antibacterial effects against pathogenic species including *S. aureus* and *L. monocytogenes*.

In summary, polyphenols exhibit a broad spectrum of antimicrobial activity [245,246,247]. This characteristic renders them promising candidates for use as antimicrobial agents [248,249,250].

### 5.4. Antidiabetic Activity

From the past to present, natural products have played an important role in human health [251,252]. In particular, plants have been widely used in different societies to combat medical conditions and diseases [253,254]. For this reason, it is being investigated how plant-derived substances can be utilized in the treatment of type 2 diabetes mellitus (T2DM), which is common with the changing lifestyle today [255,256]. Long-term metabolic condition T2DM is a defect in blood glucose regulation, which is determined by increasing blood glucose levels brought by hyperglycemia, insulin resistance, amyloid deposition, and pancreatic beta-cell malfunction [257,258,259]. According to studies conducted so far, mechanisms associated with insulin regulation, such as protecting pancreatic cells, reducing cell apoptosis and supporting cell proliferation, alleviate oxidative stress, activate insulin signaling, stimulate insulin secretion, inhibit glucose absorption, regulate gut microbiota, and modify the inflammatory response [16,260]. Therefore, dietary polyphenols play an important role in the treatment of T2DM [261,262]. In addition to these situations, It has been exhibited that some types of polyphenols, including resveratrol, curcumin, and quercetin, take charge in the reduction in inflammation and oxidative stress by changing the various signaling pathways that are related with insulin [18,263].

Various research studies demonstrate the antidiabetic activity of tea polyphenols on experimental diabetes [264,265]. Male albino rats were exposed to a fraction of green tea polyphenols [264]. Male albino rats were divided into three groups, the first group being the control group, and were initially supplemented with equal amounts of glucose. The distilled water was given only to the control group, and the other group 2 and group 3 rats were supplemented with GTP. As a result of the study, administration of GTP 500 mg/kg suppressed the increase in serum glucose level at 60 min. In animal studies, polyphenols of *Syzygium zeylanicum* L. (*S. zeylanicum* L.) demonstrated antidiabetic activity in overfeeding and high glucose-induced 2.5–3-month-old diabetic zebrafish. According to a study, animals were divided into four different groups, including normal fish, diabetic fish, diabetic fish treated with metformin (20 µM) (METFO), and diabetic fish treated with *S. zeylanicum* L. As a result of the study, polyphenols of this plant could regulate gene expression of lipid and glucose metabolism, and effect glucose uptake, utilization. Also, it can lead to normal levels of fasting blood glucose levels [266]. Different animal studies demonstrated the antidiabetic activity of *Linum usitatissimum* on 8–12-week-old female rats. Female rats were divided into three different groups of ten rats, each including normal, diabetic control, and diabetic rats. All presented results of the study exhibited a reduction in blood glucose levels and weight loss. Additionally, histopathological investigations were performed and demonstrated that plant extract partially improved the pancreas, liver, and kidney [267]. A study conducted by Zuo et al. purchased purple speckled kidney beans (*Phaseolus vulgaris* L.) used to understand antidiabetic role in the 5–6-week-old male rates [268]. After 1 week of adaptive feeding, the normal group was determined. Apart from the normal group of rats fed a standard diet (73.5% corn starch, 20% wheat bran, 5% fish meal, 1% semolina, and 0.5% sodium salt), the remaining rats with T2DM were randomly divided into five groups and fed a high-fat diet (53%, 51% corn starch, 14.6% wheat bran, 3.6% fish meal, 0.73% semolina, 0.56% sodium salt, 1.2% cholesterol, 5.8% egg yolk powder, 10% sucrose, and 10% sucrose 10% lard). After 4 weeks of feeding, rats were fasted without water for 12 h (overnight), and blood samples were collected to determine fasting blood glucose (FBG) ≥ 11.1 mmol/L. According to the study results, *Phaseolus vulgaris* L. complex proved that it can regulate blood glucose and blood lipid levels and alleviate insulin resistance in rats with T2DM. It was also found that it can alleviate the damage to pancreatic and liver tissue caused by T2DM and has the potential to restore the biological balance of intestinal microflora by increasing the concentration of short-chain fatty acids (SCFA) in the intestines of rats.

Another in vitro study including human liver and myoblast cells aimed at analyzing the antidiabetic activity of green and yellow Papaya (*Carica papaya*) [269]. In conclusion, extract of *Carica papaya* exhibited a fat-lowering effect, stimulating glucose activity in liver cells and thus possessing a potential role in antidiabetic activity. Additionally, papaya extracts stimulated diabetes-related wound healing by effecting cell migration. An in vitro study demonstrated that herbs of *Aerva lanata* L. (*A. lanata*) *Juss* possess antioxidants, anti-inflammatory, and apart from this, antidiabetic capacities. This study provided the antidiabetic role of *A. lanata* by α-glucosidase and α-amylase inhibition assays, which are related enzymes with glucose absorption and metabolism [270]. Other research included in vitro and in vivo investigations of antidiabetic activity of ethanolic propolis extract [271]. In the in vitro step, similar α-glucosidase and α-amylase inhibition assays were performed, and these experimental analyses mightily suggest that propolis may be a good choice for managing diabetes. In the study, the animals were divided into four different subgroups of five rats each. According to the experimental design of the study, diabetic rats received a 0.5 mL/100g dose from 15% or 30% propolis extract for 4 weeks, and the result of the study showed that blood glucose levels of the propolis treated group decreased. Another study aimed at investigating the effects of using vinegar extract obtained from Zhenjiang aromatic vinegar as a potential agent in the treatment of diabetes examined how this vinegar extract helped improve diabetes symptoms in mice [272]. Vinegar extract has been shown to increase body weight, lower blood sugar levels, improve glucose tolerance and insulin tolerance, and reduce inflammation in the liver. Additionally, this extract has been shown to regulate the gut microbiota and influence the levels of SCFAs. Considering the results, it is revealed that vinegar extract may play a potential role in the treatment of diabetes, and this effect may occur through modulation of the intestinal microbiota.

In a study conducted in 2020, the aim was to examine the pharmacokinetic properties of curcumin isolated from the *Curcuma longa* (*C. longa*) plant and to reveal the potential effects of this compound in the combat with diabetes mellitus disease [273]. To achieve this, molecular simulations were performed to identify different diabetes target proteins of curcumin, including SGLT-2 (PDB ID: 2DH4), PPAR (PDB ID: 3GSI), alpha-glucosidase (PDB ID: 3W37), DPP-4 (PDB ID: 2G63), and alpha-amylase (PDB ID: 1OSE). Curcumin showed strong interactions with the active sites of proteins such as DPP-4, alpha-amylase, alpha-glucosidase, PPAR, and SGLT-2 in ligand–receptor interactions. Considering the results, curcumin emerged as a strong candidate for diabetes treatment in in silico analyses and molecular docking simulations.

A study conducted by Narayanankutty et al. aimed to compare the antioxidant, anti-inflammatory, and antidiabetic activities of coconut (*Cocos nucifera*) and Palmyra palm (*Borassus flabellifer*) haustorium and to investigate their potential as a functional food [274]. *Borassus flabellifer* (*B. flabellifer*) exhibited higher DPPH radical scavenging, hydrogen peroxide scavenging, and lipid peroxidation inhibition capacities compared to *Cocos nucifera*. Evaluation of α-amylase and α-glucosidase inhibitor activities revealed that *B. flabellifer* exhibited higher activity than *Cocos nucifera*.

In general, the antidiabetic activity of different polyphenol species has been studied in vitro and in vivo, and positive results have been shown, providing that various polyphenol can show antidiabetic activity [275,276,277,278]. However, human studies are insufficient and the potential for future treatment needs to be explored.

### 5.5. Skin and Hair Health

The largest organ in the body is skin, which totally interacts with the environment and other external elements impacting its morphology and function [279,280]. Skin is a complicated organ and performs multiple physical functions, thus providing protection from environmental damaging elements such as harmful sun rays and microbes, extreme temperature, etc., by covering the complete part of the body [281]. Because of these extensive damaging elements and various skin functionalities, particular skin diseases are common. The hair follicle, a skin appendage, is a complex structure [282]. Hair, originating from specialized tissues called hair follicles on the skin, undergoes a cyclic process known as the hair cycle, consisting of three phases: anagen, catagen, and telogen. Disruption in this cycle, such as thinning, lead to conditions that trigger hair loss. Hair loss affects the balance between an individuals’ quality of life and psychosocial well-being [283]. In addition to symptoms like social anxiety and depression, hair loss can also cause psychological effects such as lack of confidence and dissatisfaction with life, making psychosocial functioning challenging [280]. 

Currently, genetics, infections, hormonal issues, stress, and mental problems are factors that are linked to skin and hair diseases. Therefore, people have used different drugs or gained attention in cosmetology for combating diverse harmful skin-related factors and treating skin-hair diseases. Hyperpigmentation, aging, allergies, tumors, and sunburn are harmful things that lead to the destruction of healthy skin tissues; however, some drugs or cosmetic products have limitations and drawbacks to treat skin problems [280]. Thus, researchers investigate less hazardous natural materials like plant-based products, and especially polyphenols are bioactive molecules of plants and they are potentially effective to reduce inflammation, carcinogenesis, hyperpigmentation, oxidative stress, aging, and pathogens [284]. In this way, skincare products should center upon improving skin appearance and protecting from diverse skin issues.

A research study aimed to understand skincare effects encapsulated in grape seed extract, and used primary human dermal fibroblast and normal human melanocytes cells [138]. According to the observations of the study, grape seed extract possesses anti-aging, brightening, and hydration-promoting roles. As a consequence of the study, grape seed extract promoted youthful appearance by inducing collagens, elastin, and fiber formation. Additionally, UVB-induced inflammation and DNA damage was reduced due to the anti-inflammatory capacity of extracts, with the help of the skin-brightening effect of grape seed extract, melanin production decreased, and by promoting hydration, the visibility of wrinkles was alleviated. Research including both in vitro and in vivo experiments aimed at investigating the anticancer and wound healing role of *Caralluma europaea* (*C. europaea*) extracts. By using cancer cell lines of human leukemia and hepatocellular carcinoma, the anticancer activity of *C. europaea* extract was tested. Additionally, in Wistar male rats, the wound-healing role of extracts was analyzed, and as a consequence of the study, wounds of rats closed after 21 days of topical applications of *C. europaea* extracts [285].

To understand the antioxidant activity of *Acacia nilotica* (*A. nilotica*) on skin health, an in vitro study was performed [286]. In this study, ethanolic extract of *A. nilotica* demonstrated powerful scavenging free radical activity due to its hydroxyl group, and thus a consequence of this study is the suggestion that *A. nilotica* is a potential natural antioxidant source [287]. A different study conducted by Montenegro et al. included human experiments to demonstrate resveratrol-loaded lipid nanocarriers’ skin hydration effect. As a consequence of this study, 20 different female volunteers performed a topical application of resveratrol-loaded lipid nanocarriers, and this application improved skin hydration, and lipid nanocarrier technology could have effective implementations in topical applications.

Another study including both in vitro antioxidant assays and in vivo skin retention experiments in male rats, and demonstrated that sunscreen cream offers a high SPF value despite having low dermal toxicity [288]. This research demonstrated that nanoparticle polyphenols and other sunscreen ingredients can combine to provide not only antioxidant effects, but also significant UVR protection. As a consequence of the in vitro antioxidant assay, curcumin, naringenin, and naringenin nanoparticles’ antioxidant capacities were tested, and naringenin nanoparticles had higher antioxidant capacity than others. In addition to in vivo skin retention experiments results, application of 2 mg/cm^2^ sun cream containing naringenin exhibited appreciable skin retention after 4 h of applications.

A research study analyzed curcumin-loaded nano cubosomal hydrogel bioactivity, including anti-irritation and antibacterial properties [289]. An in vivo skin irritation experiment was performed on shaved rats. Rats are shaved one day before starting the application, and application of hydrogel was carried out for 7 days. After 7 days, any skin irritation like redness, erythema, or edema were observed. In addition, in vitro antibacterial analysis was performed, zones of inhibition were measured against *E. coli*, and were particularly higher compared to pure curcumin. This developed strategy of curcumin-loaded cubosomal hydrogel increases the stability of curcumin, thus improving antibacterial activity when applied topically. Furthermore, a different study including lyophilized hydrophilic extracts of *Rhus coriaria* (*R. coriaria*) to investigate its wound healing and antimicrobial role [290]. By animal experiments, the wound healing effect of this plant’s extract is proven, and collagen genesis on the wound area is stimulated. Additionally, the antibacterial role of *R. coriaria* aqueous extract was demonstrated against different bacteria including *S. aureus*, *S. aureus*, *Streptococcus aureus*, *P. aeruginosa*, *E. coli*, *Proteus vulgaris*, and *Shigella* spp.

Another research study that aimed at improving the antioxidant activity of *H. sabdariffa* L. calyx extracts was performed with a liposome-containing strategy. As a consequence of this study, with the help of an in vitro antioxidant assay, the study demonstrated that liposome-containing *H. sabdariffa* L. possessed a huge antioxidative role. Also, an in vivo skin irritation experiment was performed with rabbits, in which the epidermal hair at the back area was removed, formulated, and non-formulated extracts were applied on the hairless area. At the result of the in vivo experiments, any irritation, redness, or edema were observed. This study suggests, for the anti-aging concept of skincare products, that this plant extract is precious for its topical application ability [291].

An animal study, including UV-induced pig, demonstrated that polyphenols of Acerola (*Malpighia emarginata* DC.) possess a skin-lightening effect on ultraviolet B-induced pigmentation of the guinea pig [292]. In addition to this, the plant extract reduced melanogenesis in the UV-damaged cell; therefore, melanin production decreased. Moreover, a different study was performed with strawberry-based cosmetic formulation to highlight the protective role of strawberry against UVA-induced damage on human dermal fibroblast [293]. As a result of the study, strawberry application is a possible protective agent against dermal damage caused by UV A radiation by the reduction in pro-inflammatory markers and reactive oxygen species. Additionally, another study including ethanolic extracts of *Penthorum chinense* Prush demonstrated the protective role against UVB- or H_2_O_2_-induced cell death, collagen degradation, inflammatory response, moisture loss, and oxidation on human keratinocyte cell line [294]. As a consequence, the anti-aging effect of ethanolic extracts of this plant, demonstrated by inhibiting oxidative stress induced by UVB and also ethanolic plant extracts, exhibit moisturizing effects.

A randomized double-blind human study was performed on an anti-inflammatory assay on post-adolescent women challenged with acne vulgaris. In this study, green tea supplementation demonstrated an anti-inflammatory role in acne formation, thus non-inflammatory and inflammatory lesions were recorded on the forehead, cheeks, nose, perioral area, chin, and entire face areas. As a result of this study, acne vulgaris formation and inflammation were decreased on the nose, perioral area, and chin side [295]. In an in vivo study including albino male mice wound-healing experiments, *Coffee arabica* L. was analyzed for its skin wound healing. In the study, aqueous extracts of green and roasted coffee beans were used to prepare hydrogel and were tested for their wound-healing capacity. A study has demonstrated roasted coffee bean hydrogel is less effective than the green one, thus the green coffee bean hydrogel significantly stimulates cutaneous tissue regeneration. Additionally, antioxidant and radical scavenger activities of coffee beans can control the overexposure of oxidative stress in wound areas [296].

In conclusion, regarding the effect of polyphenols on skin and hair health, there are studies about the protective role of polyphenols on skin damage in the literature. However, studies carried out in certain directions need new comprehensive research to understand and investigate new therapeutic natural agents for skin disorders.

### 5.6. Neuroprotective Effect

Regarding their potential neuroprotective activity, polyphenols are viewed as compounds that may aid neuroprotective mechanisms and potential treatments for neurodegenerative conditions, especially due to their antioxidant and anti-inflammatory effects [297,298]. The neuroprotective effects of polyphenols have focused on their ability to scavenge free radicals in the brain and their potential to reduce oxidative stress [299]. In neurodegenerative diseases including Alzheimer’s and Parkinson’s, the potential of polyphenols to neutralize reactive oxygen species and protect neurons from oxidative stress is of significant interest [299,300].

Alzheimer’s disease (AD) is a neurodegenerative disease which is a progressive and most common form of dementia in aged people, and AD is characterized by β-amyloid (Aβ) peptide aggregation formed from microtubule tau proteins. Natural polyphenols curcumin from the root of *C. longa* has multiple biological activities. Because of the antioxidant, anti-inflammatory, and anti-amyloid properties, curcumin is associated with a neuroprotective effect in AD models. Curcumin-loaded lipid-core nano capsules used in an AD study, rats injected with cur nano capsules changed behavior when triggered with Aβ peptide spontaneously in 8 min. Additionally, curcumin decreased the synaptotoxicity triggered by amyloid peptide aggregation because of its antioxidant activity, and hyperphosphorylation of tau proteins was inhibited by cur nano capsules [301]. A research study including purification of *Phyllanthus emblica* L. pomace’s polyphenols was conducted to understand antioxidants and potential anti-Alzheimer’s effects [300]. In the experimental design of the study, these bioactivities of polyphenols were measured before and after the purification step, and the result presented that the polyphenol contents of the purified extract were 2.1 times higher than that of the non-purified ones. Also, the antioxidant activity of the purified pomace’s polyphenols was improved, and purified polyphenols showed that they importantly attenuated the paralysis level of the transgenic *Caenorhabdits elegans* model of Alzheimer’s disease. In the transgenic mouse model of AD, curry spice curcumin was used to analyze its antioxidant and anti-inflammatory role in AD [302]. Ten-month-old female and male mice were tested with high and low doses of curcumin. According to the result of the study, curcumin can suppress inflammation of AD by reducing interleukin-1 and performing an antioxidative role. Also, a low dose of curcumin potentially possess a reducing effect on soluble amyloid protein [302]. A mouse model of an AD study demonstrated a combination of polyphenols to improve AD [303]. The combination of polyphenols included food-grade resveratrol, grape juice, and concord grape juice, and these combinations demonstrated reduced brain amyloid neuropathology. Therefore, this suggests that a combination of polyphenols can improve cognitive impairment in AD mouse models [303]. Sirt 1 gene modulates some biological pathways including inflammation, metabolism, and apoptosis in AD. Decreasing Sirt1 gene expression has been shown to lead to amyloid aggregation and thus potentially trigger the occurrence of AD [304]. In a research study including the activation of the Sirt1 gene via resveratrol in a rat model of AD, orally administered resveratrol significantly improved Sirt1 gene expression [305], and therefore enhanced memory function, glutathione, and antioxidative enzymes like superoxide dismutase. In conclusion, this research suggests that resveratrol may activate Sirt1 signaling and thus have a neuroprotective role on AD. Amyloid beta-induced AD mice were treated with a resveratrol, and as a result of the study, exhibited that resveratrol possesses an anti-amyloidogenic role and can improve memory function [306]. A different study including aluminum chloride induced AD rats and treatment with the polyphenols of the leaves of grapes. As a consequence of the results, the polyphenols of the leaves of grapes have an antioxidative, anti-inflammatory, and neurotrophic support role in improving brain function in AD rats [307].

Another progressive neurodegenerative disease is Parkinson’s disease (PD), which is characterized by loss of dopaminergic cells and dopamine production [308]. Also, aggregation of α-synuclein leads to the creation of lesions, which causes neuronal death. α-synuclein, which is a 140 amino acid protein, and increases in this protein cause an impaired function in the mitochondria, and this non-toxic structure leads to neuronal loss [309]. In an in vitro study performed using an inhibition procedure using the polyphenol of green tea, which is epigallocatechin-3-gallate (EGCG), it was found that it can strongly inhibit the aggregation of α-synuclein and toxicity by its antioxidant capacity [310,311,312]. Additionally, EGCG protects the neurotransmitter of dopamine, which is found in Substantia Nigra. Reduced dopamine levels are associated with the disability of movement control, so studies about the treatment of Parkinson’s diseases include the administration of green tea polyphenols, such as catechin, which can improve motor impairment and recover dopamine levels [313]. Also EGCG, in an animal study, can modulate striatal antioxidants, superoxide dismutase, and catalase to prevent dopamine disruption [314]. Another one is that the pre-treatment of EGCG reduced neurotoxicity and α-synuclein gene expression. Therefore, it can prevent apoptosis of neurons and provide antioxidant capacity [315]. In the transgenic Drosophila model of PD, different doses of curcumin (25, 50, and 100 µM) were administered in the PD flies. According to this study, the survival rate of male flies was measured with a different dose of curcumin, and the study demonstrated that curcumin significantly increased the life span of PD flies [316]. Another study including human cell lines challenged with PD was studied to understand the antioxidative and anti-inflammation roles of tea polyphenols [317]. As a consequence of the study, tea polyphenols have an active role in protection of dopamine levels by its antioxidant role. An in vitro model PD study aimed to show that unique mixes of polyphenols have a neuroprotective role [299]. In this study, researchers demonstrated that a novel mixture of polyphenols and other micronutrients, also known as A5+, possess a reducing effect against the detrimental process of PD. A5+ reduces the release of pro-inflammatory cytokines, inhibits apoptosis mechanisms, and decreases oxidative stress. As a result, the study suggests that these mixtures of polyphenols may be a therapeutic agent against PD. A rotenone-induced cellular and animal model of a PD study was performed to understand the antioxidant capacity of a nanosheet polyphenolic fraction of propolis to improve PD [318]. As a result of the study including both in vivo and in vitro experiments, the formation of nanosheets have been demonstrated to exhibit more effective antioxidant properties in PD models. 

A research study conducted by Rosa et al. demonstrated the neuroprotective effect of Olive polyphenols in in vivo *Caenorhabditis elegans* (*C. elegans*) PD models [319]. A study was performed on the lifespan, swim behavior, and heat stress assays to understand the protective role of olive polyphenols. As a result of the study, polyphenols of olives can improve the lifespan of PD models and beneficially affect the locomotion ability of PD models.

Huntington’s disease is dominantly inherited and is linked with abnormalities in the Huntingtin protein, which is a 350 kDa protein that includes glutamine repeats, but in disease conditions, there is abnormal polyglutamine expansion [320]. In the Drosophila model of Huntington’s disease (HD) is a devastating neurodegenerative disease, and nano encapsulated curcumin improves the median survival life of HD flies. Curcumin also acts as an antioxidant because it can suppress oxidative stress in diseased conditions [321]. Another Drosophila model-based study demonstrated that curcumin can suppress polyQ-mediated photo neuron degeneration and internal eye dysmorphology. Also reduced pigment loss of eye and locomotor dysfunction, which are related with polyQ aggregation in a Huntington’s disease model [320]. Similar studies have also been carried out [322,323]. 

A study about the role of curcumin on migraine patients, patients consumed curcumin as a randomized control trial study [324]. According to the result of the study, the frequency of headache attacks was decreased in the curcumin consumed group. Also, due to anti-inflammatory activity of curcumin, tumor necrosis factor-α (TNF-α), which has a role in increasing inflammation, was decreased in the curcumin administered group. In addition to this study, diverse studies about nano-curcumin administration effect on patients challenged with migraine and outcomes are the same with other research [325,326].

In general, polyphenols have a neuroprotective role on neurological disorders, and studies demonstrated that both in vivo and in vitro studies provide the finding that some polyphenols exhibit a neurotherapeutic effect because of their antioxidant or anti-inflammatory capacity; however, what is behind the mechanism of these capacity is not well known, and the bioavailability of the polyphenols is still a debated issue. Therefore, more research needs to be performed to understand or figure out all of the issues related to the interaction between neurological diseases and polyphenols.

### 5.7. Anti-Tumor and Anticancer Activity

For many years, researchers have been drawn to investigating the anti-tumor and anticancer properties of polyphenols [327,328,329]. These natural compounds demonstrate chemo preventive effects against various types of cancer [330,331]. Studies indicate that polyphenols can play significant roles, including inhibiting tumor growth and preventing cancer development, through their antioxidant, anti-inflammatory, and antiproliferative properties [332,333,334,335]. 

There are several methods to assess the antiproliferative, anti-tumor, and anticancer effects of polyphenols [336,337,338,339]. One such approach involves conducting in vitro studies utilizing cancer cell lines [340,341,342]. Through the utilization of polyphenols sourced from diverse origins or employing varying concentrations of polyphenols, researchers can investigate their impact on cancer cells, as well as their influence on cell growth and proliferation [343,344,345]. A recent study conducted by Zhang et al. investigated the inhibitory effects of polyphenols extracted from *Cerasus humilis* (*C. humilis*) on liver cancer HepG2 cells, colon cancer HCT116 cells, and stomach cancer BGC823 cells [346]. The findings revealed that *C. humilis* fruit, known for its richness in polyphenols, exhibited a significant inhibitory activity against liver, colon, and stomach cancer cells. Furthermore, key anti-tumor targets, including TP53, MAPK3, MAPK1, RELA, AKT1, PIK3R1, and 16 other genes, were identified. Another recent study revealed that the phenolic composition of *C. europaea* extracts exhibited anticancer activity against human leukemia (K562 and HL60) and liver cancer (Huh-7) cell lines [285].

In the study conducted to discover an efficient purification method for the polyphenols of *Pinus koraiensis* (*P. koraiensis*) pinecones, it has been revealed that purified polyphenols exhibit antiproliferative effects on the seven different cancer cell lines [347]. It has demonstrated higher sensitivity in the human colon cancer stem cell line (LOVO cell line) according to 3-(4,5-Dimethylthiazol-2-yl)-2,5-Diphenyltetrazolium Bromide (MTT) assays. It has been discussed that the purified polyphenols obtained in this study could be used to produce functional foods. In another study conducted by Yi et al., it was revealed that purified polyphenols of *P. koraiensis* pinecones had an anti-tumor effect on colon cancer cells by inducing apoptosis through the activation of caspases [348]. In another study, polyphenols were extracted and characterized from *P. koraiensis* bark. In this study, the effects of isolated polyphenols on colon cancer cells HT29, breast cancer cells MFC-7, stomach cancer cells BGC-823, and cervical cancer HeLa cells were investigated [349]. According to the results of the study, it was revealed that the polyphenols obtained showed the highest inhibitory effect on colon cancer cells. It is also thought that it may increase programmed cell death in cancer cells by increasing the number of apoptosis cells. In addition to their direct effects on cancer cells, the utilization of various polyphenols to treat the side effects of cancer was investigated [337,350,351].

Another approach to observe these effects is through conducting in vivo studies using animal models [341,352]. These studies typically involve monitoring tumor growth and progression in afflicted animals through the administration of polyphenols via various methods [352,353]. Comprehensive studies may encompass both in vivo and in vitro investigations [354,355]. These studies serve to elucidate the specific molecular pathways modulated by polyphenols [342,356,357]. One of the primary objectives of such studies is to examine the effects of polyphenols on apoptosis, cell cycle regulation, angiogenesis, and metastasis [358,359,360]. In a study conducted in 2021, the aim was to determine the in vitro and in vivo anti-colon cancer activity of polyphenols extracted from *Hippophae rhamnoides* L. (*H. rhamnoides* L.) [358]. This study examined the effect on colon cancer growth by assessing changes in miRNA expression profiles, cell cycle, and apoptosis. Polyphenols extracted from *H. rhamnoides* L. were purified using macroporous resins and designated as HPs60. HPs60 was characterized using Liquid Chromatography Mass Spectrometry, revealing a high kaempferol content. The anti-colorectal cancer effect of HPs60 was investigated through in vitro and in vivo studies. In the in vitro studies, human colon cancer cell lines HCT116, HT29, and FHC were utilized to evaluate the effects of HPs60 on cell viability. The results indicated a significant decrease in cell viability with increasing doses of polyphenols, demonstrating an inhibitory effect of HPs60 on cancer cell proliferation. Additionally, the alteration of miRNA expression profiles induced using HPs60 treatment contributed to the observed changes in cell viability by regulating cell cycle progression and apoptosis. In vivo studies on mice suggested no apparent toxicity during HPs60 treatment, as evidenced by the absence of significant differences in bodyweight between groups. Conversely, there was a significant reduction in tumor volume after HPs60 treatment compared to the control group, indicating its anticancer properties in reducing tumor growth in vivo. Furthermore, HPs60 treatment was shown to affect the expression of specific microRNAs (miRNAs) in tumor-bearing mice. In conclusion, the study underscores the promising anti-tumor properties of HPs60, suggesting its potential for further clinical investigations in colorectal cancer treatment. 

Similarly, in another study exploring the biological activity, antioxidant, anti-tumor, and immune-modulating properties of anthocyanins and polyphenols extracted from blueberries (*Vaccinium* spp.), both in vivo and in vitro experiments were conducted [361,362]. The study evaluated the antioxidant activity and tumor proliferation-inhibiting properties of a mixture of anthocyanins and polyphenols, as well as purified and crude samples obtained from in vitro experiments [361]. This assessment aimed to establish a correlation between the antioxidant capacity of different extracts and their ability to inhibit tumor growth. The antioxidant activity of the purified samples was notably higher than that of the crude extracts. In vivo experiments of the study investigated the effects on tumor growth in mice with breast cancer based on MDA-MB-231 cells. According to the findings, the blueberry anthocyanin and polyphenol crude extract mixed group exhibits the most potent tumor suppressor effects, likely attributable to synergistic interactions among the compounds. Moreover, it was observed that the extract also enhanced the general health status of mice by increasing cellular immune function, boosting antioxidant enzyme activity, and reducing lipid peroxidation.

In conclusion, numerous studies suggest that polyphenols may exert an active role in the prevention of cancer development, modulating disease progression, and potentially enhancing treatment modalities including radiotherapy and chemotherapy [353,363,364,365,366].

### 5.8. Other Effects

The impact of polyphenols on health extends across various dimensions [367,368]. For instance, a study examined the effects of supplementing antihypertensive therapy with dietary flavonoids on blood pressure, lipid profile, body mass index, leptin, and C-reactive protein (CRP) levels in hypertensive patients [369]. According to the findings, a notable decrease in both systolic and diastolic blood pressure, along with reductions in total cholesterol, LDL cholesterol, and triglyceride levels, was observed following the incorporation of dietary flavonoids. Simultaneously, upon assessment of body mass index and leptin levels, it was evident that the inclusion of dietary flavonoids in the regimen could yield favorable outcomes concerning issues such as obesity. In addition to these functions, a significant decline in CRP levels was noted, implying a potential role in mitigating the risk of cardiovascular disease.

Another study aimed to determine the effects of antimicrobial activity of some polyphenols including apigenin, catechin, luteolin, morin, myricetin, naringin, quercetin, and routine flavonoids on the strains most found in dental plaques that potentially trigger detrimental dental health outcomes [370]. For this purpose, *Candida albicans* fungal strain, *Streptococcus oralis* (*S. oralis*), *E.coli*, *Actinomyces viscosus* (*A. viscosus*), *E. faecalis*, *Streptococcus sanguinis* (*S. sanguinis*), *Actinomyces naeslundii* (*A. naeslundii*), *Agreggatibacter actinomycetemcomitans*, *Lactobacillus casei*, and *S. aureus* strains were used. According to the results, especially Rutin, quercetin, and morin showed antibacterial activity against *A. viscosus* and *A. naeslundii*. Although each flavonoid exhibits antibacterial properties against some strains, no antibacterial effects have been demonstrated against *S. sanguinis* and *S. oralis*.

In a study conducted by Bogolitsyn et al., the aim was to determine the effects of polyphenols obtained from the brown algae *Fucus vesiculosus* on human lymphocytes, monocytes, and neutrophilic granulocytes [204]. The polyphenols increased the number of adhesive leukocytes in the blood of both healthy individuals and leukemia patients. Additionally, the leukocytes from leukemia patients showed a lower tendency to adhere to surfaces compared to those from healthy individuals, indicating that algal polyphenols dose-dependently modulated the adhesive activity of leukocytes. Additionally, the polyphenols enhanced the adhesion and interaction abilities of cells by activating defense pathways against tumor cells.

**Table 1:** Health benefits of isolated polyphenols.

Health Benefits	Polyphenols From	Type of Polyphenols	Outcome	References
Antioxidant activity	*Rhododendron tomentosum*	Rosmarinic acid Caffeic acid Chlorogenic acid Rutin Quercetin	- Exhibit DPPH radical scavenging activity	[185]
	Rye Bread	*	- Exhibit an improvement in functional and nutritional value	[166]
	Red cabbage	*	- Exhibit scavenge free radicals activity	[171]
	Herbal tea	Gallic acid Catechin Caffeic acid Ferulic acid Epicatechin Gallate Quercetin Kaempferol	- Brewing time affects the extractability of polyphenols and antioxidant activity in tea	[164]
	*Fabacea*	*	- Exhibit hydrogen peroxide and nitric oxide scavenging activities	[188]
	*Rosa roxburghii*	Gallic acid Ellagic acid Gallocatechin Epigallocatechin Catechin Epicatechin	- Exhibit scavenge free radicals activity	[178]
	*	3,4-dihydroxyphenylacetic acid Homovanillic acid Vanillic acid Caffeic acid Gallic acid Phloroglucinol Pelargonidi Ellagic acid	- Exhibit anti- or pro-oxidants activity - Exhibit hydroxyl radicals scavenging activities	[153]
	De-oiled rice bran	Vanillin Ferulic acid Sinapic acid Chlorogenic acid	- Exhibit an enhancement in vitro digestion - Exhibit anti-inflammatory activity	[181]
	Corn bran	4-hydroxybenzaldehyde *p*-coumaric Sinapic acid Ferulic acid		
	*Amaranthus lividus*	*	- Exhibit scavenge free radicals activity	[183]
	Banana	3-Hydroxyphenylpropionic acid Ferulic acid Caffeic acid Anthocyanins Cyanidin 2′-Hydroxyformononetin Quercetin Neoeriocitrin Scopoletin 2′-Hydroxyformononetin	- Exhibit capturing free radicals and antioxidant activity	[187]
	*Zhourat*	Gallic acid	- Exhibit higher antioxidant capacity	[186]
	*Leptospermum scoparium*	*	- Exhibit scavenge free radicals activity	[190]
	*Sambucus ebulus*	Chlorogenic acid Caffeic acid glucoside 3-*p*-coumaroylquinic acid 3-*p*-Feruloylquinic acid Catechin Epicatechin Procyanidin Kaempferol Quercetin Piceid	- Exhibit DPPH radical scavenging activity	[191]
	*Rubus* spp.	Gallic acid Neochlorogenic acid Procyanidin Catechin Vanillic acid Caffeic acid Epicatechin *p*-coumaric acid Quercetin Ferulic acid Kaempferol	- Exhibit bioavailability and bioactivity	[119]
	*Thymus serpyllum* L.	Rosmarinic acid Luteolin Salvianolic acid	- Exhibit antibacterial activity	[157]
	*Euphorbia antisyphilitica*	*	- Exhibit an inhibition of lipid oxidation	[168]
	Herbal tea and green tea	*	- Exhibit scavenge free radicals activity	[172]
	*Eugenia uniflora* leaves *Eucalyptus microcorys* leaves *Myrciaria cauliflora* seeds	Ellagic acid Kaempferol Quercetin Myricetin 2,3-Di-*O*-galloyl-glucose 2,3,6-Tri-*O*-galloyl-glucose 1,2,3,4,6-Penta-*O*-galloyl-glucose 4,6-*O*-HHDP-glucose Gemin Oenothein Isocoriariin Tellimagrandin Pedunculagin Tellimagrandin Eugeniflorin Camptothin Oenothein	- Exhibit antioxidant capacity	[152]
	*Satureja hortensis* L.	Rutin Rosmarinic acid	- Exhibit radical scavenging activity - Exhibit an improvement the lipid peroxidation process	[169]
	*Chamerion angustifolium*	Oenothein Quercetin Myricetin Luteolin Kaempferol Gallic acid Chlorogenic acid *p*-coumaric acid Ellagic acid Benzoic acid etc.	- Exhibit antioxidant capacity	[167]
	*Sargassum wightii*	Gallic acid Quercetin Ferulic acid Vanillin	- Exhibit higher antioxidant capacity	[154]
	*Ulva rigida*			
	*Gracilaria edulis*			
	*Pistacia lentiscus* L.	Feruloylquinic acid *p*-coumaroylquinic acid 5-*O*-caffeoylquinic acid Monogalloyl glucose Gallic acid 5-*O*-galloylquinic acid Chlorogenic acid Digalloylquinic acid Procyanidin Epicatechin Catechin Epigallocatechin gallate Trigalloylquinic acid *p*-coumaric acid Myricetin Quercetin Kaempferol Luteolin Apigenin	- Exhibit higher antioxidant capacity	[165]
	*Amaranthus dubius*	2-O- Caffeoylglucaric acid Ferulic acid 4-Hydroxycinnamic acid Kaempferol Caffeoylquinic acid Myricetin Quercetin	- Exhibit higher antioxidant capacity - Exhibit anti-inflammatory activity	[179]
	*Amaranthus spinosus*	Dihydromyricetin Ferulic acid 4-Hydroxycinnamic acid Feruloylquinic acid Kaempferol Caffeoylquinic acid Myricetin Quercetin		
	*Amaranthus tricolor*	2-O-Caffeoylglucaric acid Ferulic acid 4-Hydroxycinnamic acid Kaempferol Caffeoylquinic acid Myricetin Quercetin		
	*Amaranthus viridis*	Ferulic acid 4-Hydroxycinnamic acid Myricetin Quercetin Quercetin		
	Carrot	Gallic acid Protocatechuic acid Vanillic acid 4-hydroxybenzaldehyde	- Exhibit scavenge reactive oxygen species activity - Exhibit antioxidant capacity	[158]
	*Echinacea Purpurea*	Caftaric Chicoric acids Catechins	- Exhibit antioxidant capacity	[159]
	*Malus domestica* borkh	Chlorogenic acid *p*-coumaric acid Quercetin -3-O-galactoside -3-O-arabinoside Phloretin-2′-O-glucoside Catechin Epicatechin Procyanidin	- Exhibit higher antioxidant capacity	[184]
	*Eucalypts* leaf	*	- Exhibit antioxidant capacity - Exhibit scavenge free radicals activity - Modulate gut microbiota	[182]
	*Nigella sativa* L.	Gallic acid Hydroquinone Apigenin Naringenin Quercetin Kaempferol Rutin	- Exhibit antioxidant capacity	[180]
	*Ipomoea batatas*	Cyanidin Peonidin Pelargonidin	- Exhibit antioxidant capacity	[163]
	*Vitis vinifera* L.	Flavan-3-ol Proanthocyanidin Anthocyanins	- Exhibit antioxidant capacity	[193]
	Coffee silverskin	Caffeoylquinic Feruloylquinic acids	- Exhibit an improvement bioaccessibility	[129]
	Coffee	*	- Exhibit antioxidant capacity	[189]
	*Polyscias fruticosa* roots	*	- Exhibit antioxidant capacity - Exhibit scavenge free radicals activity	[170]
	*Chroogomphus rutilus*	Protocatechuic acid	- Exhibit higher antioxidant capacity - Exhibit anti-inflammatory activity - Exhibit cytotoxic effect	[160]
Anti-inflammatory activity	*Tetraclinis articulata*	*	- Exhibit antioxidant capacity - Exhibit scavenge free radicals activity	[215]
	*Pleurotus ostreatus*	Cathechin Sinapic acid Resveratrol etc.	- Exhibit antioxidant activity	[216]
	Green tea and red wine	*	- Exhibit antioxidant activity	[217]
	*Punica granatum* L.	Luteolin Rosmarinic acid Quercetin Eriodictyol etc.	- Exhibit antioxidant capacity	[371]
	*Thymus vulgaris*	Rosmarinic acid Luteolin etc.		
	*Rosmarinus officinalis* L.	Chlorogenic acid Caffeic acid etc.		
	*Echinacea purpurea* L.	Ellagic acid Gallagic acid etc.		
	*Maclura tricuspidate* *Pyrus Montana Naka*	Gallic acid Protocatechuic acid Chlorogenic acid *p*-hydroxybenzoic acid Vanillic acid Caffeic acid Rutin ρ-coumaric acid Ferulic acid Rosmarinic acid Salicylic acid Quercetin Cinnamic acid Taxifolin	- Exhibit antioxidant activity - Modulate iNOS, COX-2, and TNF-α protein expression	[214]
	Olive Oil	Oleacein Oleocanthal	- Exhibit scavenge free radicals activity - Modulate the synthesis of pro-inflammatory lipid mediators	[197]
	Finger millet	Protocatechuic acid Catechin Chlorogenic acid Naringin	- Exhibit anti-obesity effect - Modulate production of cytokines - Exhibit antioxidant activity	[218]
	Kodo millet	Catechin Naringin *p*-coumaric acid Taxifolin Ferulic acid Sinapic acid Methyl vanillate		
	*Rhamnus prinoides* L’Herit	Caffeic acid Protocatechuic acid Kaempferol Gallocatechin Proanthocyanidin Luteolin Quercetin Apigenin Rutin etc.	- Exhibit antioxidant activity	[200]
	*Petroselinum crispum* *Apium graveolens* *Coriandrum sativum*	*	- Exhibit scavenge free radicals activity - Exhibit membrane stabilizing effect	[206]
	Huangjiu	Protocatechuic acid Catechin Chlorogenic acid Vanillic acid Caffeic acid Syringic acid *p*-coumaric acid Ferulic acid Sinapic acid Rutin Quercetin	- Modulate production of cytokines	[210]
	*Arabidopsis thaliana*	Caffeic acid Quercetin Kaempferol Synapic acid Luteolin	- Modulate production of cytokines	[196]
	*Ilex latifolia*	Quinic acid Caffeoylquinic acid Shikimic acid Rutin Hyperoside etc.	- Exhibit scavenge free radicals activity - Exhibit antioxidant activity - Modulate production of cytokines	[211]
	*Cynara scolymus* L.	Hydroxytyrosol Verbascoside Apigetrin Oleuropein Quercetin Pinoresinol Apigenin	- Exhibit antioxidant activity	[205]
	*Acalypha hispida*	Gallic acid Quercetin Ellagic acid *p*-coumaric acid etc.	- Exhibit antioxidant activity - Modulate inflammatory pathways	[195]
	*Lonicera caerulea* L.	Chlorogenic acid Caffeic acid Catechin Epicatechin Cyanidin etc.	- Modulate production of cytokines	[202]
	*Prunus domestica* L.	Chlorogenic acid *p*-coumaric acid Rutin etc.	- Exhibit antioxidant activity - Exhibit induction of lipid peroxidation	[203]
	*Gaultheria procumbens* L.	Protocatechuic acid Caffeoylquinic acid *p*-hydroxybenzoic acid Vanillic acid Catechin Epicatechin *p*-coumaric acid Procyanidin Quercetin Kaempferol etc.	- Exhibit antioxidant activity	[201]
	*Baccaurea ramiflora* Lour	Rosmarinic acid	- Exhibit antioxidant activity - Modulate production of cytokines	[194]
	*Libidibia ferrea* *Parapiptadenia rigida* *Psidium guajava*	Catechin Gallic acid	- Exhibit inhibition character on bacterial zone	[212]
	*Phaseolus vulgaris* bean	Sinapic acid Ferulic acid Naringenin Catechin Quercetin etc.	- Exhibit antioxidant activity - Modulate production of cytokines	[199]
	*Verbascum phlomoides*	Gallic acid Rosmarinic acid Caffeic acid Ferulic acid Quercetin etc.	- Exhibit antioxidant activity	[198]
	*Rubus coreanus* Miquel	*	- Exhibit anti-super bacterial activity - Modulate production of cytokines	[209]
Antimicrobial activity	*Guizotia abyssinica* L. leaf and flower extracts	Tannins Glycosides Flavanoids Phenols	- Exhibit antioxidant activity	[225]
	*Retama monosperma*	Flavonoids Tannins Quinones Anthocyanins	- Exhibit antioxidant activity - Exhibit antimicrobial activity against *Staphylococcus aureus*, and *Bacillus cereus*.	[3]
	*Filipendula ulmaria*	Quercetin Rutin	- Exhibit higher antioxidant activity - Exhibit antimicrobial activity against *Listeria monocytogenes*	[223]
	*Salvia officinalis*	Quercetin Apigenin Naringenin Rutin		
	*Rosmarinus officinalis*	Luteolin Eriodictyol		
	*Sideritis scardica*	Quercetin Rutin Epicatechin		
	*Geranium purpureum*	Quercetin Rutin Catechin Epicatechin Hydroxytyrosol		
	Banana peels	*	- Exhibit higher antioxidant activity - Exhibit antimicrobial activity against *Staphylococcus aureus*, *Pseudomonas aeruginosa*, *Escherichia coli*, and *Saccharomyces cerevisiae*	[240]
	*Artemisia aucheri*	*	- Exhibit an improvement in health effect in mice against *Campylobacter jejuni*	[143]
	Grape pomace	Anthocyanins Phenolic acid Flavonoids Stilbenes	- Modulate antioxidant activity - Exhibit an increase in *Lactiplantibacillus plantarum* growth - Exhibit antimicrobial activity against *Escherichia coli, Bacillus megaterium,* and *Listeria monocytogenes*	[243]
	*Alcea rosea*	Gallic acid Salicylic acid Pyrogallol Cinnamic acid Catechin Naringin Ferulic acid	- Exhibit antimicrobial activity against *Escherichia coli*	[142]
	*Achillea millefolium*	Salicylic Succinic acids Folic acid Caffeic acid Kaempferol Luteolin Apigenin and other phenolic and flavonoid compounds	- Improve the average daily weight gain, food intake, liver function, and antioxidant status - Decrease the ileum population of *C. jejuni* in the mice challenged by *C. jejuni* infection	[140]
	*Rheum ribes*	Gallic acid Salicylic acid Caffeic acid Cinnamic acid Catechin Ellagic acid Ferulic acid	- Exhibit antimicrobial activity against *Escherichia coli* and improve health parameters of mice	[141]
	*Lycium chinense* Mill.	Quercetin Kaempferol Catechin Flavan-3-ols Coumaric acid Chlorogenic acid Procyanidin	- Exhibit antibacterial activity against *Bacillus subtilis* and *Proteus vulgaris*	[224]
	Propolis	*p*-coumaric acid Ferulic acid Chrysin	- Exhibit scavenge free radicals activity - Exhibit antimicrobial activity against *Escherichia coli*, *Bacillus subtilis spizizenii nakamura*, and *Candida albicans*	[219]
	*	Combined polyphenols	- Exhibit protective effect against influenza A and SARS-CoV-2	[234]
	*Punica granatum* L.	Ursolic acid Corosolic acid Arjunolic acid	- Exhibit antimicrobial activity against *Staphylococcus aureus*	[221]
	*Hibiscus sabdariffa* L.	Kaempferol Cyanidin Quercetin	- Exhibit antimicrobial activity against *Candida albicans, Staphylococcus aureus*, and *Listeria monocytogenes*	[244]
	*Spirulina*	*	- Exhibit antimicrobial activity against drug resistant food pathogens.	[367]
	*Zhourat*	Gallic acid etc.	- Exhibit inhibition character on bacterial zone	[186]
	*Lantana camara* L.	Tetramethylhexadec-2-en-1-ol Linolenic acid 2,6-Dimethoxyphenol 9,12-Octadecadienoic acid	- Exhibit scavenge free radicals activity - Exhibit antimicrobial activity against *Xanthomonas axonopodis* pv. *glycines* and *Xanthomonas oryzae* pv. *oryzae*	[173]
	*Picea abies* L. *Larix decidua Mill* *Pinus sylvestris* L. *Pseudotsuga menziesii* *Juniperus communis* L.	Gallic acid *p*-coumaric acid 2,5-dihydroxybenzoic acid 4-hydroxybenzoic acid Chlorogenic acid Caffeic acid Syringic acid Vanillic acid Sinapic acid Ferulic acid Salicylic acid Cinnamic acid Vitexin Apigenin Kaempferol Luteolin Quercetin Naringenin Rutin	- Exhibit antioxidant and antiradical activity - Exhibit antimicrobial activity	[230]
	*Natural polyphenols*	TF3 TF2b TF1 TF2a Hesperidin EGCG Myricetin Quercetagetin	- Exhibit inhibition effect on the RNA-dependent RNA polymerase of SARS-CoV-2	[235]
	*	Apigenin Catechin Luteolin Morin Myricetin Naringin Quercetin Rutin	- Exhibit inhibition effect on bacterial and fungal growth	[370]
	Olive oil	*	- Exhibit antibacterial activity against *Listeria monocytogenes* - Exhibit a reduction in intracellular ATP concentrations	[227]
	*Prunus dulcis*	Epicatechin Catechin	- Exhibit the inhibition character against bacterial growth and reducing expression of viral proteins	[236]
	*Vitis vinifera* L.	Gallic acid Coumaric acid Vanillic acid Chlorogenic acid Cyanidin Catechin Caffeic acid Peonidin 3-O-glucoside Epicatechin Luteolin Resveratrol Ferulic acid	- Exhibit antioxidant activity - Exhibit inhibition character on bacterial zone	[233]
	*Moringa oleifera*	Coumaric acid Myricetin Quercetin Kaempferol Resveratrol Naringenin Biochanin A Naringin Catechin	- Exhibit antioxidant activity - Exhibit inhibition character on bacterial zone	[232]
	Olive oil	*	- Exhibit antimicrobial activity against *Cronobacter sakazakii* - Exhibit a reduction in intracellular ATP concentrations - Exhibit an increase in cell membrane permeability	[231]
	*Achillea* *pachycephala* *Achillea millefolium* *Achillea nobilis* *Achillea filipendulina* *Achillea santolina* *Achillea aucheri*	Chlorogenic acid Caffeic acid Quercetin Luteolin Rutin Ferulic acid	- Exhibit antioxidant activity - Exhibit antibacterial activity against *Staphylococcus aureus*, *Bacillus cereus*, *Escherichia coli*, *Staphylococcus epidermidis*, and *Salmonella typhimurium*	[220]
	*	Stilbenes Cinnamic Benzoic Flavonoids Coumarins Naphtoquinones	- Exhibit antimicrobial activity against *Staphylococcus aureus*, *Bacillus subtilis*, *Listeria monocytogenes*, *Escherichia coli*, *Pseudomonas aeruginosa*, and *Salmonella Enteritidis*	[242]
Antidiabetic activity	*Solanum anguivi*	*	- Exhibit antioxidant activity - Exhibit scavenge free radicals activity	[263]
	*Syzygium zeylanicum* L.	Gallic acid Catechin Epicatechin Caffeine Quercetin Apigenin Ethyl gallate Rutin Ellagic acid Chlorogenic acid Quercitrin	- Exhibit antidiabetic activity by modulation of gene expressions of lipid and glucose metabolism	[266]
	*Cucumis dipsaceus*	Rutin Gallic acid	- Increase phenolic and flavonoid compounds - Exhibit scavenge free radicals activity	[278]
	*Phaseolus vulgaris* L.	*	- Exhibit antidiabetic activity, improve the T2DM outcomes	[268]
	*Gracilaria bursa-pastoris*	Gallic acid Catechin 4-hydroxy benzoïc acid Chlorogenic acid Caffeic acid Syringic acid Vanilline *p*-coumaric acid Sinapic acid Quercetin 7,3′,4′-flavon-3-ol Naringin Rutin Salicylic acid Quercetin Cinnamic acid Luteolin Apigenin Kaempferol Flavone Flavanone	- Exhibit antioxidant activity	[255]
	*Carica papaya*	*	- Exhibit fat-lowering effects and stimulate glucose activity in liver cells	[269]
	*Curcuma longa*	Curcumin	- Exhibit pharmacokinetic activity	[273]
	*Cocos nucifera*	Gallic acid Ferulic acid 4-Hydroxycinnamic acid *p*-coumaric acid Quercetin	- Exhibit DPPH radical scavenging activity - Exhibit α-amylase and α-glucosidase inhibitor activities	[274]
	*Borassus flabellifer*	Gallic acid Ferulic acid 4-Hydroxycinnamic acid Quercetin Myricetin-3-O-glucoside		
	Vinegar extract	4-Hydroxybenzoic acid Ferulic acid Salicylic acid Vanillic acid Protocatechuic acid Catechin Ellagic acid Gallic acid Gallocatechin 3-O-gallate Rutin etc.	- Exhibit an improvement in glucose tolerance and insulin tolerance - Exhibit a reduction in inflammation in the liver	[272]
	*Vigna radiata* L.	Gallic acid Vitexin	- Modulate gut microbiota - Exhibit antioxidant activity - Exhibit anti-inflammatory activity	[368]
	*Quercus suber* *Quercus ilex* *Quercus coccifera* *Quercus canariensis*	Chlorogenic acid	- Exhibit α-amylase inhibitory activity	[260]
	Red wine	Gallic acid Caftaric acid Coutaric acid Malvidin 3-O-glucoside Petunidin 3-O-glucoside	- Exhibit anti-radical effect - Exhibit antioxidant activity	[298]
	*Aerva lanata* L. Juss	Gallic acid Protocatechuic acid Caffeic acid Syringic acid 4-hydroxybenzoic acid Vanillic acid Gentisic acid Sinapic acid *p*-coumaric acid Ferulic acid Rosmarinic acid Isoferulic acid Salicylic acid	- Exhibit antioxidant activity	[270]
	*Linum usitatissimum*	*	- Exhibit reduction in blood glucose levels, weight loss, also possess recovery role for pancreas and liver	[267]
	*Vigna unguiculata*	Gentisic acid Coumaric acid Ferulic acid Quercetin	- Exhibit antioxidant capacity - Exhibit an inhibition of α-glucosidase and α-amylase activities	[275]
	*Lonicera caerulea* L.	Cyanidin Quercetin Chlorogenic acid Flavan-3-ol Catechin Epicatechin	- Exhibit antioxidant activity - Exhibit an inhibition of α-glucosidase and α-amylase activities	[276]
	*Propolis*	Protocatechuic acid Catechin Caffeic acid Syringic Acid Epicatechin *p*-coumaric acid Ferulic acid Luteolin	- Exhibit antioxidant capacity - Exhibit an inhibition of α-glucosidase and α-amylase activities	[271]
	*Rosmarinus officinalis* L.	*	- Exhibit antioxidant activity - Exhibit anti-aging activity	[137]
	*Lagerstroemia speciosa*	Caffeic acid Ellagitannins Flavonoids Quercetin	- Exhibit a decrease in fasting blood glucose, body weight, levels of serum biomarkers, tissue weight, and body fat	[253]
	Peanut shell	Luteolin Pyrogallol Catechol Phloroglucinol Quercetin	- Exhibit protective effects against diabetes - Exhibit a reduction in fasting blood glucose levels	[16]
Skin and hair effects	* Caralluma europaea *	Luteolin Gallic acid Hesperetin Quercetin Myricetin Ferulic acid Salicylic acid Naringenin	- Exhibited the improving role for wound healing and by a reduction in hepatocellular carcinoma perform anticancer activity	[285]
	*Vitis vinifera* seed	*	- Exhibited anti-aging, brightening, and hydrating effects. Also, this effect could be increased by encapsulation	[138]
	* Rhus coriaria *	Anthocyanins Flavonoids Phenols Hydrolyzable tannins Gallic acid Quercetin	- Exhibited that plant extract possesses antibacterial and wound healing properties	[290]
	* Penthorum chinense * Prush	*	- Exhibited anti-aging activity by protection from UVB ray, reduction in free radicals, and increase skin moisture	[294]
	*	Naringenin Curcumin	- Exhibited antioxidant effect by decreasing dermal toxicity and also appreciable skin retention effect observed	[288]
Neuroprotective activity	Propolis	*	- Exhibit antioxidative role on PD model	[318]
	*	Mix of polyphenols	- Exhibit reducing effect against the detrimental process of PD and release of pro-inflammatory cytokines, inhibits apoptosis mechanisms, and decreases oxidative stress	[299]
	*Phyllanthus emblica* L.	Gallic acid Epicatechin Ethly gallate Chebulagic acid Ellagic acid Quercetin	- Exhibit anti-Alzheimer’s effect and antioxidant capacity in transgenic Alzheimer model	[300]
	*	Curcumin	- Exhibit improving the median survival life of HD flies and also acts as an antioxidant agent by suppressing oxidative stress	[321]
	*Olive*	*	- Exhibit improving lifespan of PD models and beneficially affect locomotion ability	[319]
	*	Resveratrol	- Exhibit enhanced memory function, glutathione, and antioxidative enzymes by stimulating Sirt1 gene expression	[305]
	*	Curcumin and fatty acid	- Exhibit reduced effect on gene expression of pro-inflammatory cytokines, thus relaxing symptoms	[325]
	*	Curcumin	- Exhibit possess suppressing role on polyQ mediated photo neuron degeneration and reduce locomoter dysfunction	[322]
	Grape leaves	*	- Exhibit antioxidative, anti-inflammatory, and neurotrophic effect to support improving brain function	[307]
	Tea	*	- Exhibit protection role for dopamine levels by its antioxidative and anti-inflammatory role	[317]
	*	Curcumin and fatty acids	- Exhibit decreasing role on interleukin-6 serum levels and decrease in symptoms	[372]
Anti-tumor/Anticancer	*Cuminum cyminum*	*	- Exhibit inhibitory activity against colon, lung, and breast cancer cell lines - Exhibit antioxidant capacity	[29]
	*Cerasus humilis*	*	- Exhibit inhibitory activity against liver, colon, and stomach cancer cells	[346]
	*Caralluma europaea*	Kaempferol Luteolin Trans-ferulic acid Syringic acid	- Exhibit anti-tumoral activity against the human leukemic and liver cancer cell lines	[285]
	*	Isoeugenol	- Exhibit anti-proliferative, anti-apoptotic, and anti-migrative against breast cancer cells	[332]
	*Camellia sinensis*	Epigallocatechin-3-gallate	- Exhibit antithrombotic, antitumor, and antiangiogenic activities	[350]
	*	Quercetin Fisetin	- Exhibit inhibition of cell proliferation - Exhibit induction of reactive oxygen species formation	[330]
	*Viscum album*	Epicatechin Quercetin	- Exhibit an apoptotic-like effect	[373]
	Apple	Cyanidin-3-O-arabinoside	- Exhibit anti-tumoral activity against the human colon cancer cell line - Exhibit an inhibitory effect on proliferation - Exhibit induction of cell apoptosis	[334]
	*Hippophae rhamnoides*	Sinapinic acid Ferulic acid Coumaric acid 7-Hydroxycoumarine Kaempferol 5,7-Dihydroxy-2-(4-hydroxy-3-methoxyphenyl)-4-oxo-4H-chromen-3-yl-6-O-(6-deoxy-α-L-mannopyranosyl) hexopyranoside	- Exhibit anti-tumoral activity against the human colon cancer cell line - Modulate miRNA expression profiles	[358]
	*Artemisia argyi* leaf	Neochlorogenic acid Chlorogenic acid Cryptochlorogenic acid Isochlorogenic acid	- Exhibit inhibitory effects on cervical and colon cancer cell lines - Exhibit antioxidant activity	[335]
	*Ziziphus jujuba*	*	- Exhibit inhibitory effects on colon cancer cell lines - Exhibit an inhibitory effect on proliferation	[340]
	*Coriandrum sativum* L.	Flavonoids Catechins Rutin	- Exhibit cytotoxicity against the leukemic cell lines	[336]
	*Empetrum nigrum*	*	- Exhibit strong antioxidant activity - Exhibit high antibacterial potential - Exhibit an inhibition of cell proliferation and induction of apoptosis	[329]
	*Pinus koraiensis* bark	Penta-hydroxy flavone	- Exhibit antioxidant capacity - Exhibit antiproliferative effects - Exhibit scavenge free radicals activity	[349]
	*Sabal yapa* leaves	Tricin Luteolin Apigenin	- Exhibit antioxidant capacity - Exhibit potent anticancer effects against Ehrlich ascites carcinoma cells	[352]
	Sugarcane	*	- Exhibits an inhibition of cell proliferation and induction of apoptosis	[331]
	*Varthemia candicans* *Peganum harmala* *Suaeda vermiculata* *Conyza dioscoridis*	*	- Exhibit cytotoxicity against the human hepatocellular carcinoma cells	[328]
	*Euphorbia lathyris*	Esculetin Euphorbetin Gaultherin Kaempferol	- Exhibit inhibitory effects on colon cancer cell lines - Exhibit antiangiogenic capacity	[359]
	*Ipomoea batatas*	Caffeic acid	- Exhibit chemo-sensitizing effects	[343]
	*Vaccinium* spp.	Pelargonidin-3-O-galactoside Delphinidin-3-glucoside Chlorogenic acid isomers Epicatechin gallate Malvidin-3-O-glucose Kaempferol-3-rhamnoside Hexose ferulic acid esters Myricetin-3-O-hexose	- Exhibit antioxidant capacity - Exhibit antiproliferative effects - Exhibit inhibitory effects on breast cancer	[361]
	*Thalassia testudinum*	*	- Exhibit inhibitory effects on colon cancer cell lines	[341]
	*Eugenia involucrata*	Gallic acid Catechin *p*-coumaric acid Rutin Myricetin Quercetin	- Exhibit antioxidant capacity - Exhibit anti-tumoral activity in a pancreatic cancer cell line	[339]
	*Agrimonia pilosa*	Agrimoniin	- Modulate activation of mitochondria-dependent apoptosis - Exhibit cytotoxicity against the cervical cancer cell line	[344]
	Peanut skin	Proanthocyanidin-B2	- Exhibit antiproliferative effects	[354]
	Extra-virgin olive oil	Oleacein	- Exhibit anticancer activity against the cutaneous melanoma - Modulate miRNA expression profiles	[365]
	*Cinnamomum cassia*	*	- Exhibit anticancer activity against the colon cancer cell lines	[366]
	*Vaccinium macrocarpon*	Cyanidin Peonidin	- Exhibit anticancer activity against the colon cancer cell lines	[353]
	*Camellia sinensis*	Epigallocatechin	- Exhibit inhibitory effects on breast cancer	[337]
	Green tea	Epigallocatechin	- Exhibit inhibitory effects on human lung cancer cells	[342]
	Foxtail millet Bran	Vanillic acid Glucosyringic acid Ferulic acid 4-hydroxybenzoic acid Vanillic acid Syringic acid *p*-coumaric acid Vitexin Ferulic acid Isoferulic acid Biferulic acid 4,4′-dihydroxy-3,5′-dimethoxy,3′-bicinnamic acid	- Exhibit inhibitory effects on colon cancer cell lines - Modulate gut microbiota	[362]
	*	Tannic acid	- Exhibit cytotoxicity against the glioblastoma cells - Exhibit antiglioma activity	[356]
	*	Resveratrol Pterostilbene	- Exhibit an inhibition of tumor growth	[357]
	Olive oil	Oleacein	- Modulate cell cycle arrest and apoptosis	[360]
	*Caesalpinia spinosa*	*	- Exhibit anti-tumor effects against breast and melanoma tumor - Modulate cell cycle arrest and apoptosis	[327]
Other effects	Green tea Dehydrated red delicious apple Dark chocolate	*	- Exhibit antihypertensive - Exhibit inhibition of cholesterol absorption - Exhibit inhibition of endothelial lipoprotein lipase - Exhibit a reduction in C-reactive protein plasma levels	[369]
	*Fucus vesiculosus*	*	- Exhibit anti-radical activity - Exhibit scavenge free radicals activity	[204]

* Not identified.

## 6. Polyphenols in Nutritional Aspect

It has been found that consuming polyphenol-rich foods can help support health, primarily due to their antioxidant properties, as well as their anti-inflammatory, anti-carcinogenic, and other functions [298,374]. Additionally, these properties are thought to help protect gut health by promoting the growth of beneficial bacteria [368]. These features encourage the consumption of polyphenol-rich foods [375]. Incorporating these foods into the diet enriches the nutrition of infants, children, young adults, the elderly, and athletes, resulting in various positive effects [376,377,378].

### 6.1. Maternal and Infant Health

Early life is an important period in which the infant gut microbiome is formed. The development of the gut microbiota in infancy and early childhood can influence how health and potential diseases are shaped later in life [379]. Disruptions in the gut microbiota during this period trigger the development of chronic diseases including allergies, asthma, and obesity both in childhood and adulthood [380,381,382]. Research has shown that human milk contributes to the development of the infant’s gut microbiota and is an important source [376]. Therefore, the mother’s breastfeeding behavior protects the baby against respiratory, gastrointestinal, and intestinal pathogens, while also shielding against the risks of inflammatory deterioration [383]. In this case, it is thought that the microbial factors in the mother are transferred to the infant through human milk and that the transfer of non-microbial molecules also occurs. For this reason, studies explaining the relationship between diet and gut microbiota in adults have tried to establish a relationship between postpartum mothers [384,385,386,387]. The diet of postpartum mothers, the changes made in this diet, and the amount of food types consumed affect the microbiota of the mother and thus change the human milk microbiome. This, in turn, may shape the gut microbiome of the infants [384].

Polyphenols, molecules with many properties vital for plant defense, are abundant in the human diet. Polyphenols derived from a variety of food sources have beneficial effects on multiple metabolic disorders, cognitive impairment, or protection against disorders such as cancer and aging. Among them, positive effects including antioxidant, anti-inflammatory properties, hormonal, and mitochondrial function regulation have the potential to improve maternal milk production and breastfeeding performance. The type and pattern of the mother’s diet is important for the health of the baby not only during pregnancy, but also during breastfeeding before and after this period [388]. For this reason, a Mediterranean Diet (MD)-type diet, rich in fiber and polyphenols, supports the mother’s nutritional composition and is valuable for both the mother’s health and the infant’s development [389]. There are few studies carried out by dairy animals (such as goat and cow) which demonstrated that polyphenols of Fenugreek (*Trigonella foenum-graecum* L.) increase the yield and quality of milk and milk fat concentrations [389,390,391,392]. *Trigonella foenum-graecum* L. is the most commonly used to increase the quality of milk supply in post-partum women [393]. Plants contain diverse types of polyphenols like quercetin, rutin, isovitexin, diosgenin, vitexin, and saponins [394]. Also, other studies show that *Trigonella foenum-graecum* L. has an important role in increasing milk flow and yield, oxytocin expression, and fat concentration on pregnant Sprague–Dawley rats [395,396]. 

Researchers have demonstrated that Moringa (*Moringa oleifera*), which has a variety of flavonoids, including kaempferol, myricetin, quercetin, and phenolic acid, also affects the milk concentration and increases macronutrients of milk quantity including protein and fat in dairy animals [397,398]. Additionally, a researcher, Olvera, carried out a study on dairy animals with the same plant. According to the result of the study, there is no effect on milk yield or quality [399]. in vitro studies demonstrates that *Moringa oleifera* plant’s extracts or leaves can prevent oxidative stress, reduce ROS, and increase the glutathione levels and gene expression of casein in bovine mammary epithelial cells. Therefore, *Moringa oleifera* plants have a protective role against induced oxidative stress in vitro [400,401]. 

Moreover, other herbal mixture formulas, including *Sauropus androgynus*, *Trigonella foenum-graecum* L., and *Moringa oleifera*, were tested for their lactation-stimulating activity on lactating rats [402,403]. The result of the study showed that milk yield increased. Additionally, other animal studies, which are Balb/c mice and female rats, exhibited the lactation hormone stimulating activity of *Sauropus androgynus* and milk thistle (*Silybum marianum*) [404,405]. According to the results of these studies, gene expression of the prolactin hormone, which is associated with the increasing of mammary milk production in post-partum women, and oxytocin hormone, which is also known as the milk flowing (milkejecting) hormone, were increased [406,407]. Together with these studies, Sani et al. conducted the effect of the *Launaea taraxacifolia* plant’s polyphenol resveratrol on milk yield and serum prolactin and oxytocin levels in rats [408]. The results of the study have proven that resveratrol has the potential to increase milk yield and prolactin and serum oxytocin levels.

There are two different studies which are about the protective role of quercetin in rodents. One of these studies demonstrated that quercetin polyphenols provide increased prolactin production in the pituitary gland, and the other one demonstrated that the same polyphenol molecule from *Ligustrum lucidum* plant can reduce mammary gland inflammation [409,410]. A different study conducted by Zhao et al. showed the activity of citrus peel extract on lactation [411]. According to the results of the study, the yield of milk is increased in dairy animals.

A study by Sanchez et al. investigated the effect of a diet rich in fiber and polyphenols supplemented during pre-pregnancy, pregnancy, and post-pregnancy breastfeeding at the intestinal level in both mothers and infants [389]. In the study on pregnant rats and their infants, this diet was shown to have a trophic effect in both pregnant rats and their infants. However, further studies are needed to elucidate the mechanisms behind these effects and to establish a more accurate relationship.

### 6.2. From Childhood to Elderly

The consumption of polyphenol-rich foods is important for individuals of all ages, including children, adults, and the elderly [297,412]. Incorporating polyphenol-rich foods can support growth and development in children and adolescents [413]. Additionally, polyphenols can be consumed to enhance cognitive function and general health, particularly in adults and elderly individuals [414,415,416]. Furthermore, for adults and the elderly, adding polyphenols to the diet can help protect general health, reduce the risk of chronic diseases, and support cardiovascular health [417,418].

In a study conducted by Ziauddeen et al., data from the National Diet and Nutrition Survey Rolling Programme (NDNS RP) 2008–2014 were examined to assess (poly)phenol intake in the United Kingdom population [413]. This included the intake of flavonoids, phenolic acids, and stilbenes from foods consumed by participants. The aim was to determine the main (poly)phenols and their amounts consumed among different age groups (children and adults) and genders (men and women). Children were classified into three age groups: 1.5–3, 4–10, and 11–18 years. Adults were classified into four age groups: 19–34, 35–49, 50–64, and 65 years and older. While gender representation was equal in the child groups, there were more female participants in the adult groups. The study results indicated that (poly)phenol intake increased with age, with this increase being generally higher in male participants. Among children, the primary sources of (poly)phenols were fruit juice, potatoes, legumes, and tea. For adults, the main sources of (poly)phenols were tea, chocolate, wine, fruits, and vegetables. 

Childhood obesity is associated with numerous health problems. In a study conducted in 2019 with obese children aged 8–10 years, the aim was to investigate the relationship between childhood obesity and bone disorders by focusing on osteoclastogenesis in obese children and adolescents [377]. The results of the study revealed that sweet cherry polyphenol extracts inhibited spontaneous osteoclastogenesis observed in obese children. The extracted polyphenols inhibited osteoclastogenesis by reducing the formation of multinucleated TRAP+ osteoclasts in peripheral blood mononuclear cell (PBMC) cultures obtained from obese children in a dose-dependent manner. Additionally, the polyphenol extracts reduced the ability of PBMCs to form large resorption areas on calcium phosphate film-coated Millenium slides, thereby inhibiting the bone resorption activities of osteoclasts. Considering the expression of pro-osteoclastogenic cytokines, it was found that TNFα mRNA levels were significantly decreased. On the other hand, when the effects of polyphenol extracts on cell viability in peripheral blood mononuclear cell cultures were evaluated using the MTT test, it was observed that the polyphenol extracts were non-toxic and supported the maintenance of healthy cells. According to the study’s results, sweet cherry extracts rich in polyphenols may help prevent and/or improve bone health problems associated with obesity. 

Another study aimed to examine the effects of a flavonoid-rich blueberry drink on children’s cognitive functions, specifically targeting cognitive benefits in children aged 10 years [419]. To evaluate the effects on general cognitive performance after blueberry drink consumption, several tasks were administered, including response inhibition (Go-NoGo test), response interference (Stroop test), visual memory (N-back test), and object location (Object Location task). No significant differences were found in these tasks. Following Rey’s Auditory-Verbal Learning Test, participants were administered short (2 min) and long (25 min) delayed recall tests after consuming a blueberry drink. Participants who consumed the blueberry drink demonstrated better performance compared to those who consumed a placebo drink, particularly in long delay recalls. When evaluating the results, it was observed that blueberry anthocyanins had positive effects on certain memory functions in children, although this effect did not extend to all cognitive areas.

In a study conducted with 400 children between the ages of 4 and 12, the aim was to investigate the relationship between the amount of dietary polyphenols consumed by children and the risk of Attention Deficit Hyperactivity Disorder (ADHD) [420]. To examine the protective role of polyphenols against ADHD, researchers investigated the antioxidant effects of polyphenols, their potential to increase cerebral blood flow, and their effects on the regulation of neurotrophic factors. It has been suggested that polyphenols may be protective against ADHD through their ability to modify membrane fluidity and adrenergic receptors, exhibit antioxidant effects, induce vasodilation, and regulate catecholamine metabolism.

In a study conducted in 2016, the aim was to investigate the relationship between polyphenol intake and the risk of type 2 diabetes [421]. To achieve this objective, an observational cohort analysis was performed on nondiabetic participants, involving 3430 elderly individuals. The findings revealed that an increase in polyphenol intake was associated with a reduced risk of type 2 diabetes. In another study conducted by Guo et al., the effect of total polyphenol levels in urine samples on body weight and body mass index in an elderly population with a high cardiovascular risk was investigated [422]. The study, conducted between 2003 and 2006, included male and female participants with an average age ranging from 66.2 to 68.3 years. Participants had diseases including diabetes, hypertension, heart disease, and dyslipidemia. During the five-year follow-up period, a significant decrease was observed in the body weight and body mass index parameters of the participants. Moreover, polyphenol levels increased significantly with the consumption of foods belonging to the Mediterranean diet. Upon evaluation, the study revealed that polyphenol intake potentially reduces the risk of obesity in elderly individuals with a high cardiovascular risk. Another study conducted by Guglielmetti et al. aimed to investigate the effects of a polyphenol-rich diet on intestinal health in elderly individuals [423]. To achieve this objective, inflammation, oxidative stress, vascular function, as well as metabolomic and microbiomic profiles were examined among 50 elderly participants. The study found that a polyphenol-rich diet yielded positive effects on intestinal permeability in the elderly, resulting in decreased serum zonulin levels. Furthermore, significant reductions were observed in inflammatory markers such as CRP, IL-6, and TNF-α, oxidative stress markers including DNA damage, and indicators of vascular function. Additionally, the study revealed the role of polyphenol-rich diets in maintaining metabolomic profiles and microbiomic balance among the elderly.

### 6.3. Athlete Health

One of the beneficial actions created by the athlete’s nutritional needs is improving athlete performance. Environment, endocrine functions, muscle–fiber relationship, athlete’s goal, nutritional, and genetic factors generate differences among individuals while also potentially impacting the athlete’s performance [424]. Although a number of factors affect the success of an athlete, sports nutrition is important among them. Genetic and dietary interactions can affect the availability of nutrients and the body functions related to sports. The quantity and type of macronutrients, namely carbohydrates, lipids, and proteins, in an individual’s diet plan demonstrate a crucial relationship with athletes’ muscle functions and performance [425]. In this context, it has been shown recently that the type and quantity of protein are critical for muscle growth and athlete performance, and variations in protein intake and amino acid absorption-metabolism among individuals are linked both to protein quality and quantity, as well as to genetic differences among individuals [426]. Genetic variations can influence the amount of bioactive peptides derived from protein sources and thus affect muscle activity, growth, and their utilization. For this reason, daily nutritional advice includes different customized dietary recommendations for each athlete during training or before-during-after competition. In addition to these macronutrients, it is also recommended to consume foods rich in nutrients including manganese, butyrate, omega-3, and polyphenols on a daily basis, as well as to consider the use of supplements like antioxidants and anti-inflammatories [427]. Additionally, nutrition provides energy to the body, and it can protect physiological equilibrium. Moreover, nutrition also possesses a vital function, which is facilitating the response of the body to induced stress by exercise [428]. Therefore, an athlete needs to take charge in homeostasis of oxidative stress during training. When oxidative stress arises by the production of reactive oxygen species, and if equivalence happens between the training adaptations, it can cause inflammation, cellular damage, and inhibit muscle recovery [429,430]. During exercise, supplementation of antioxidants affects athlete performance and recovery, and mitochondrial adenosine triphosphate production is not 100% efficient, so superoxide radicals are formed. Since the more oxygen is used, the more superoxide radicals are generated that need to be extinguished; muscle damage causes excessive production of free radicals, which inhibits recovery; endogenous mechanisms for the elimination of radical species are insufficient [431]. 

Plant-based foods are attracting attention in modern sports nutrition due to their significant nutrients, enhancing recovery, and a broad range of bioactive components [432]. Especially, polyphenols can provide diverse advantages for athletes, including antioxidant, anti-inflammatory, and antibacterial properties of polyphenols, sustaining general wellness [433]. Due to these advantages, some polyphenols, including resveratrol, quercetin, and curcumin, have been associated with muscle health [434]. Currently, there are many studies on sports nutrition and polyphenols. Most of these studies include both the important effects of polyphenolic compounds on post-exercise muscle damage and their effects on improving physical performance [435]. Polyphenol species have been studied under a variety of conditions with different supplementation strategies for different durations and dose amounts [378]. Although polyphenols, which have been studied for many years due to their many beneficial effects, were initially investigated in connection with epidemiological problems, the apparent result of this connection is the inverse relationship between polyphenols and the presence of oxidative stress-related pathologies. Therefore, in order to prevent oxidative stress caused by physical activity, diets high in polyphenols have been investigated [436,437]. 

On top of these, quercetin polyphenol supplementation in athlete performance has been investigated. Quercetin is a flavonoid type of polyphenol and has a significant role in the muscle remodeling process, such as inhibition of muscle loss by the regulation of protein catabolism, while it also induces muscle anabolism by increasing phosphorylation [438]. In a study involving elite cyclists, increases in aerobic performance were observed in athletes taking 1200 mg of the supplement daily for 6 weeks [439]. Another study involving 12 young men looked for a solution to eccentric-induced muscle damage by giving them placebo and quercetin supplements [440]. As a result of the study, the group receiving 1g of quercetin supplementation daily for 14 days had fewer plasma markers of eccentric muscle damage compared to the group receiving placebo. This suggests that quercetin repairs muscle damage. In another study involving 24 female and 33 male active athletes, it was aimed to evaluate their condition after long (5, 10 km) running performance [441]. Although competition times were similar in athletes who received either placebo (728 mg maltodextrin) or 140 mg Zynamite plus 140 mg quercetin for 24 h for 1 h before competition and every 8 h thereafter, post-competition muscle soreness was reduced in the polyphenol supplemented group. This means that the combination of Zynamite and quercetin alleviates muscle soreness and damage and accelerates recovery of muscle performance. The benefits of quercetin supplementation are thought to be positively influenced when taken in high doses; however, more research is needed to confirm the optimal dosage. 

Moreover, other polyphenol resveratrols, which also affect athletic performance primarily found in grape skin and red wine, can induce the anabolic metabolism of muscles by enhancing elements of signaling pathways [426]. In the conducted study, 47 male runners were administered high-purity resveratrol supplementation in the form of pure grape juice for 14 days while employing the method of running until exhaustion [442]. According to the results of the study, the grape juice provided as a supplement improved the athletes’ exhaustion times. A different study is about the effect of grape juice on athlete performance, involving 14 runner men to analyze the capacity of aerobic exercise. In conclusion, a single dose of purple grape juice has shown an ergogenic effect in recreational runners by increasing the time to exhaustion during running and antioxidant activity [443]. Apart from these, animal studies are also included in the literature [444,445,446]. However, small sample sizes in studies on resveratrol and the use of various indefinite doses of supplements make it difficult to determine a specific range of safety/efficacy of this supplement in athletes, so more studies are needed. In addition to the benefits of resveratrol supplementation for athletes, resveratrol can probably control glucose and insulin sensitivity [447]. In athletes, it is very important that the body uses insulin as efficiently as possible during a physical transformation. A study aimed at the effect of resveratrol on glucose, as a result of the study resveratrol, can improve insulin sensitivity and glucose control in diabetic rats [448]. Therefore, these results suggest that resveratrol might be a powerful bioactive compound for athletes challenged with hyperglycemic fluctuations and insulin resistance. Furthermore, curcumin, which is a main bioactive polyphenol of the spice herb turmeric, and it especially possesses antioxidant and anti-inflammatory properties. Because of its antioxidant capacity, it can easily suppress oxidative stress and induce muscle recovery by increased myofibrillar proliferation, reducing muscle loss in the animal model of induced muscle atrophy [426]. In human studies, supplementation of curcumin performed a reduction in muscle damage and inflammation biological markers, and an approximate administration of 150–1500 mg/day pre-, post-, and during exercise might improve athletic performance and recovery of muscle by decreasing exercise-induced muscle damage and arranging the inflammation response [449,450,451,452,453]. However, research is needed on the potential possible effect of curcumin supplementation on the molecular mechanisms governing resistance-training-induced muscle gains. In addition, circumin benefits are linked to its relationship with the intestinal microbiota. In animal studies, curcumin and resveratrol exhibit anti-inflammatory and anti-carcinogenic effects on microbiota by modulating the *Firmicutes*/*Bacteroidetes* ratio [454,455]. Also, curcumin can improve the beneficial bacteria in microbiota including bifidobacteria, lactobacilli, and butyrate-producing bacteria, and promotes intestinal barrier integrity by the immunomodulatory action role [456,457,458].

A single-blind, randomized, and parallel-design clinical trial research demonstrated that a fermented grape drink hardaliye has an antioxidative role in young soccer players [459]. According to a study, there were two different groups, the hardaliye group and placebo group. In total, 250 mL/d of hardaliye drink was consumed by the participants in the hardaliye group, and other participants consumed a placebo drink during 28 days. In the results of the study, consumption of hardaliye was able to increase total serum antioxidant capacity level and decrease the oxidative stress index and the level of NO compared to the placebo group. The consumption of hardaliye in young soccer players demonstrated antioxidative effects.

A different study that includes two sub-studies, researched sugar-polyphenol rich diluted cloudy apple juice effect’s on intestinal barrier of ultra-marathon runners [460]. In this randomized double-blind study, the endurance run was carried out three times with a placebo, apple juice, and water supplementation. After the endurance run, beverages were instantly digested, and then the blood samples of the athletes were taken at five different time points. In the results of the study, the effects on the markers of intestinal permeability and inflammation in the serum of participants who consumed the test drink after physical activity was significant, and positive results were recorded compared to participants who drank the placebo drink. Diluted apple juice is well known for rehydration after physical exercise, and may also have positive effects on the intestinal barrier and immune system after exercise.

Another research study demonstrated the fatigue-relieving effect of *Lonicera caerulea* Berry polyphenols on mice, which are experimentally exhaustive swimming [421]. According to the results of the study, the formula of the polyphenol compound effectively prolonged the swimming time of mice at room and low temperature. Furthermore, accumulation of the metabolite, metabolism of energy, and down-regulated secretion of inflammatory factors were improved. Polyphenol compounds of *Lonicera caerulea* can relieve swimming fatigue at room and low temperature.

Tropospheric ozone, a component of urban air pollution, is formed through photochemical reactions involving hydrocarbons, nitrogen oxides, and volatile organic compounds. Ozone exposure also affects the central nervous system, contributing to neurological disorders such as Alzheimer’s and Parkinson’s disease, cognitive impairments, and neuroinflammation. In this context, both human and animal studies show the neurotoxic effects of ozone. These effects include the reduction in dopaminergic neurons, accumulation of pathological proteins, and so on [461]. The hippocampus, one of the brain regions, is not resistant to ozone exposure due to various factors. In this region, brain-derived neurotrophic factor (BDNF), factors involved in processes including neural growth, differentiation, memory, and learning are present. BDNF-dependent human and animal studies have proven that acute bouts of exercise stimulate neuronal function [462,463,464], brain vascularization, and neuronal synthesis through the elevation of BDNF [465], promoting better mood and improved learning. However, exercise in polluted air has been shown to inhibit acute exercise-induced BDNF secretion. Therefore, exposure to polluted air is thought to inhibit cognitive health and neural central system repair. Thus, a study on high-intensity cyclists showed that polyphenol supplementation increased BDNF levels in cyclists exercising in polluted air [461]. 

## 7. Conclusions and Future Perspective

Polyphenols, which are natural compounds, possess numerous subtypes and exhibit as wide a range of biological activities as the number and position of hydroxyl molecules they contain. Originating from plants, polyphenols are prevalent in contemporary Mediterranean and Asian diets, and researchers are interested in establishing a relationship between dietary intake and their potential health benefits. Each type of polyphenol has a distinct bioactive character, and these compounds have been the subject of extensive investigation. Their beneficial effects, widely studied for their antioxidant, anti-inflammatory, antimicrobial, neuroprotective, skin health, anticancer, and antidiabetic properties, are not yet fully elucidated. The mechanisms behind these biological effects, and how long and how many polyphenols can be shown to have these effects are among the gaps in the literature. In addition, polyphenols, which are at the forefront with their beneficial effects, are poor in terms of bioavailability and may be excreted by the metabolism before reaching the target action site after digestion or may lose their bioactivity after metabolic events. Therefore, strategies to improve the bioavailability of polyphenols have been developed and presented in the literature. Among these, liposomal and nano drug delivery strategies have been widely investigated. However, there is no definitive conclusion applicable to all types of polyphenols, indicating the need for further research. Polyphenols, which have an important place in the nutrition of individuals of all ages, including athletes, due to their beneficial effects, are the focus of interest in nutrition types. However, there are no definite and sufficient data in the literature in terms of how many each individual can consume or take as a supplement, and comprehensive research is lacking in terms of all polyphenol types. Subsequently, polyphenols known to enhance the bioavailability of their beneficial effects actually elucidate the science in the future in terms of drug development to improve metabolic diseases, topical application to skin problems, or functional foods in nutrition to improve athletic performance or health. In this context, filling the gaps in the literature and integrating polyphenols into every moment of life has the potential to increase the prevalence of healthier individuals and athletes in future generations.
